# Transferable Neural Network Potentials and Condensed Phase Properties

**DOI:** 10.1021/acs.jcim.5c01355

**Published:** 2025-09-11

**Authors:** Anna Katharina Picha, Marcus Wieder, Stefan Boresch

**Affiliations:** † Faculty of Chemistry, Institute of Computational Biological Chemistry, University of Vienna, Wien 1090, Austria; ‡ Open Molecular Software Foundation, Davis, California 95616, United States; § Vienna Doctoral School of Chemistry (DosChem), University of Vienna, Wien 1090, Austria

## Abstract

Transferable neural network potentials (NNP) are undergoing rapid development. Many practical applications of NNPs focus on single molecules; e.g., using NNPs as a fast replacement for quantum chemical methods for dihedral angle scans in force field development. Similarly, the reference data on which most transferable NNPs have been trained are single molecule properties. As NNPs are beginning to be used to simulate more complex systems, such as solute–solvent simulations, the question arises whether the current generation of transferable NNPs is accurate enough to reproduce condensed phase properties, which in most cases are outside the training domain of the models. Here we present a first analysis of how well two transferable NNPs (ANI-2x, MACE-OFF23­(S/M)) perform in reproducing properties such as density, heat of vaporization, heat capacity, and isothermal compressibility of several pure liquids (water, methanol, acetone, benzene, *n*-hexane at room temperature, and *N*-methylacetamide at 100 C). In addition, we examine selected radial distribution functions and the self-diffusion constant. We find specific weaknesses for each of the models, and seemingly small flaws lead to poor performance when applied to condensed phase simulations. The varied outcomes observed with the machine learning potentials suggest that, currently, selecting an architecture or model for all-NNP simulations of real-world applications requires careful consideration and testing.

## Graphical abstract

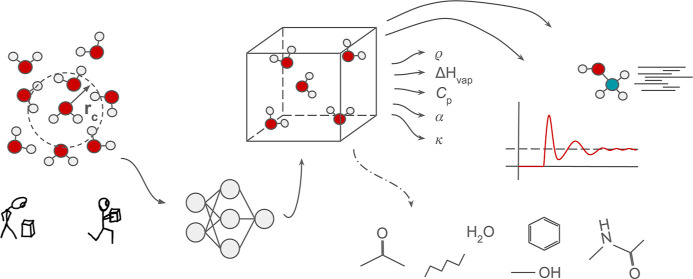

## 1 Introduction

During the past years, the field of neural network potentials (NNPs), sometimes also called machine learning (interatomic) potentials or quantum machine learning potentials, has exploded.^1–3^ These models are trained against high-level QM calculations; once trained, they can reproduce intra- and intermolecular interactions at almost the same quality as the underlying QM method at a fraction of the computational cost. Applications include conformer generation,^4^ chemical property prediction,^5^ and even free energy simulations.^6–8^ Calculations using NNPs are sufficiently fast so that they can be employed to carry out MD simulations of small to medium systems, such as water boxes or solute–solvent systems. Even simulations of proteins in water have been reported, though the computational effort is considerable.^9^

Various architectures have been proposed for NNPs, offering a broad spectrum of complexity, accuracy, and computational cost.^10^ Notable examples include SchNet,^11^ PaiNN,^12^ NequIP,^13^ SpookyNet,^14^ ANI,^15,16^ MACE,^17^ among others. However, NNPs differ not only in their underlying architectures but also in the way they are trained. The choice of a training data set and area of application suggests a differentiation between *transferable* NNPs^9,16,18,19^ and *special purpose* (*bespoke*) NNPs.^20,21^ The latter, while very accurate, can only be applied to the specific applications for which they have been trained. Transferable NNPs, on the other hand, aim to predict potential energies and forces of entire classes of molecules. Once the model has been trained, the objective is to apply it to multiple systems without need for further QM calculations, which, of course, requires very large and highly diverse training data sets. Bespoke and, in particular, transferable NNPs are mostly trained on single point energies and forces. Thus, single molecule energies computed by NNPs, such as ANI-2x,^16^ outperform force field results.^3^ Because of their speed, NNPs are used to replace high-level DFT calculations during force field development or refinement, see, e.g., ref 22.

However, it is unclear whether the current generation of NNPs and the training sets currently in use are capable of reproducing condensed phase properties, such as densities, heat of vaporization, radial distribution functions (RDFs), or (self-)­diffusion coefficients. Some investigations in this direction have been reported. Fu et al.,^23^ for example, looked at some properties of liquid water. Similarly, the StableNetGuardOwl (https://github.com/Exscientia/StableNetGuardOwl) project provides automated setup for the calculation of water RDFs, as well as thermodynamic properties of organic liquids, but no results are available yet. Recently, Ranasinghe et al.^24^ reported RDFs for water obtained with several NNPs. The description and validation of MACE-OFF23 reports water RDFs, as well as densities and heats of vaporization of water and organic liquids.^9^ Poltavsky et al. compare the performance of several NNPs when applied to materials and interfaces.^25^ Ple et al. report several condensed phase properties, with a focus on water.^26^

Checking the adequate reproduction of condensed phase properties is a routine step during classical force field development.^27–30^ Transferable NNPs can, in principle, replace classical force fields.^3^ Therefore, we decided to investigate available, ready-to-run transferable NNPs focusing on condensed phase properties. Specifically, we analyze the ability of ANI-2x^15,16^ and MACE-OFF23,^9^ two transferable NNPs which can be used within OpenMM^31^ and OpenMM/ML,^32^ to reproduce several physicochemical properties of water, methanol, acetone, benzene, and *n*-hexane at room temperature, as well as *N*-methylacetamide (NMA) at 373.15 K. In addition, we looked at RDFs and diffusion coefficients. We critically compare the performance of the NNPs to reproduce condensed phase properties, i.e., when applied outside their training domain, to that of a traditional, classical force field (FF). Specifically, we use the TIP3P model^33^ for water, and the CHARMM general force field (CGenFF)^34–36^ for the organic liquids.

The remainder of this manuscript is organized as follows: Section 2 starts with an overview of the information flow in feed forward neural networks and, specifically, the NNPs studied in this work, outlining the key steps involved. We then summarize the theoretical framework for calculating the ensemble and structural properties considered in this study. Section 3 provides the details of the investigated systems, including system sizes and computational setup, as well as the software packages used for data analysis. Section 4 presents our results, covering ensemble properties, RDFs, and self-diffusion coefficients. Finally, Section 5 summarizes our key findings and discusses the current state of the transferable NNPs investigated. In the Appendix, we present force field results as a function of cutoff radius.

## 2 Theory

### 2.1 Neural Network Potentials

NNPs as studied in this work are feed forward neural networks and can be viewed abstractly as a composition of trainable functions, or layers, that sequentially map between adjacent feature spaces. Mathematically, a feed forward network $f:X_{0}\to X_{L}$ is expressed as

$$f=f_{L}◦f_{L-1}◦\cdot \cdot \cdot ◦f_{1}$$

with

$$f_{i}:X_{i-1}\to X_{i},,i=1,...,L$$

and

$$X_{i}\subseteq R^{d_{i}},d_{i}\in N,,i=0,...,L$$

where each layer *f*_*i*_ transforms input features $x_{i-1}\in X_{i-1}$ into output features $x_{i}\in X_{i}$ . The composition of these mappings enables the network to progressively extract higher-level representations from raw inputs, making neural networks versatile tools for approximating complex functions in various domains. To apply this approach to molecular systems, these mappings need to respect mandatory symmetries of the predicted properties and require a concept of locality. For the potential energy, the mandatory symmetries are translational, rotational and permutational invariance.^21^

Since the seminal work of Behler and Parrinello,^37^ multiple architectures how to implement NNPs have been proposed; see, e.g., ref 10. As explained in Methods, for pragmatic reasons this work focuses on ANI-2x^16^ and MACE.^9,17^ The total energy of a system described by an NNP is expressed as

$$E_{total}=\sum_{i=1}^{N_{atoms}}E_{i}$$

where *E*_*i*_ represents the energy contribution of an atom *i*, derived from its local chemical environment N(i) . The local environment is defined as the set of all atoms *j* within a cutoff distance *r*_c_ from atom *i*

$$N\left(i\right)=\left\{j|d_{ij}<r_{c},j\neq i\right\}$$

with *d*_*ij*_ = ∥**r**_*i*_ – **r**_*j*_∥_2_ as the Euclidean distance between atoms *i* and *j*.

ANI-2x^16^ employs a fixed featurization strategy based on predefined radial and angular symmetry functions, inspired by the Behler–Parrinello approach.^37^ It uses predefined two-body and three-body functions based on interatomic distances and angles, ensuring that the representation is both rotationally and translationally invariant. Locality is enforced by constructing an atomic environment vector (AEV) for each atom *i*, which encodes the interactions (both two-body and three-body) with all neighboring atoms within the predefined cutoff *r*_c_.

In contrast, the MACE architecture utilizes^17^ a dynamic, learnable representation based on message passing (see Figure 1) and the atomic cluster expansion (ACE).^38^ Here, each atom is treated as a node in a graph, and the interatomic interactions are represented as edges. The process begins by initializing node features as learnable embeddings of the chemical elements. For every neighboring atom *j* (i.e., those within the cutoff *r*_c_ of atom *i*), a message *m*_*ji*_ is generated that encodes both radial and angular information—through the use of Bessel functions for radial and spherical harmonics for angular dependencies.^17^ These messages are then aggregated and used to update the node features iteratively, allowing the network to refine its representation of each atom’s local environment. This recursive update not only extends the effective interaction range beyond the initial cutoff but also provides the model with greater flexibility to learn complex interatomic interactions while preserving the necessary invariances and equivariances.

**Figure 1:**
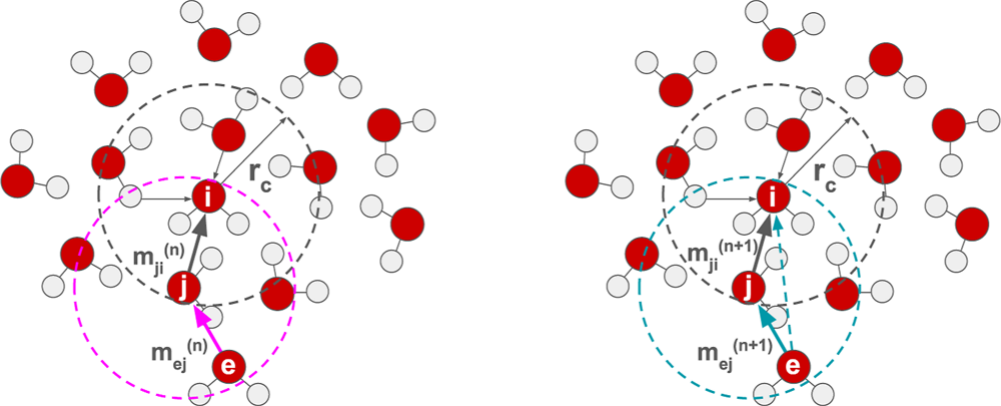
An illustration of message flows in an architecture using message passing, given cutoff radius *r*_c_. Left: In round *n*, messages from atoms within the cutoff range are collected, here illustrated for atoms j and i. Right: In round *n* + 1, the process is repeated, effectively enlarging the cutoff radius and allowing information flow from atom e to i.

**Figure 2:**
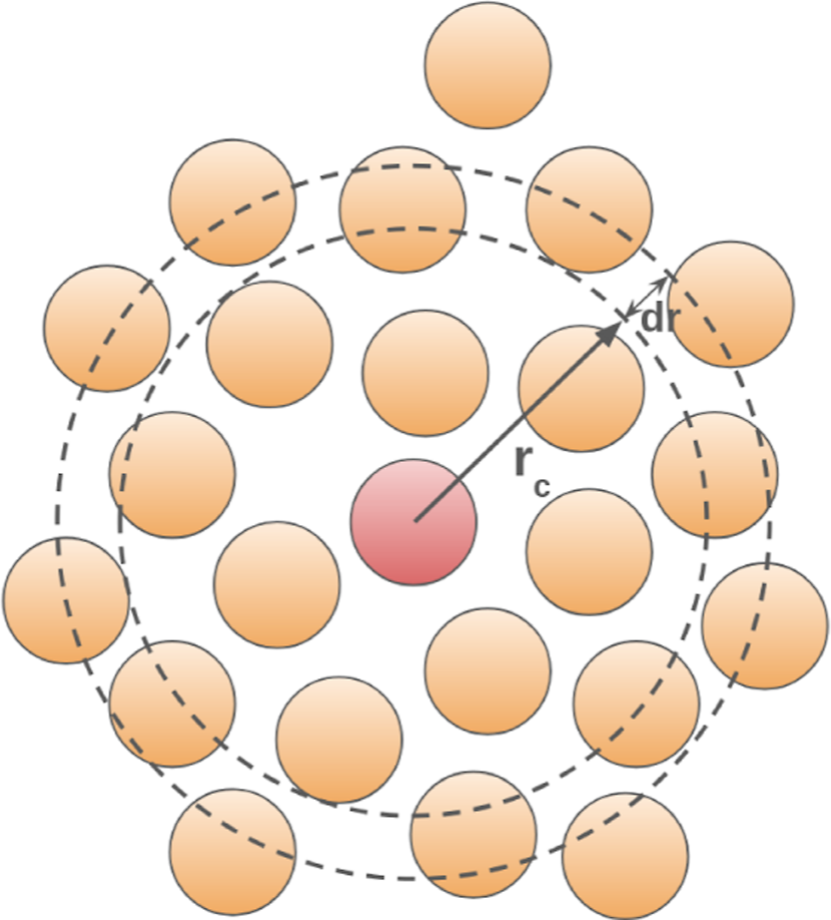
Constructing an RDF.

In addition to the architectural differences, the ANI-2x and MACE-OFF models studied in this work were trained on different data sets, in particular with respect to water. Some of the pertinent differences, as well as details concerning the treatment of interactions (cutoff radii etc.) are provided in Table 1.

**Table 1:** Training Data and Simulation Settings for the NNPs, as Well as Details of Non-Bonded Treatment in the FF Simulations

NNP simulations		
model	training data	cutoff
ANI-2x^a^	elements: H, C, N, O, S, F, Cl	radial: 4.6 Å
	data set: ANI-2x, organic molecules up to 8 non-hydrogen atoms	angular: 3.1 Å
	QM reference calculations (functional/basis set):	no message passing
	ωB97X/6-31G*	
	max. size of water cluster: 15 molecules	
MACE-OFF23^b^	elements: H, C, N, O, F, P, S, Cl, Br, I	small: 4.5 Å
	data set: SPICE version 1 ^39^ with additions, organic molecules up to 90 atoms	medium: 5 Å
	QM reference calculations (functional/basis set):	2 message updates^c^
	ωB97M-D3(BJ)/def2-TZVPPD	
	max. size of water cluster: 50 molecules	
Simulation Settings for the Classical MD (FF) Simulations^d^		
FF (CGenFF)^34–36^	treatment of nonbonded interactions:	LJ switch region:
	switched Lennard-Jones (LJ) potential	10 Å–12 Å
	PME^40^ for electrostatics	
	water model: TIP3P^33^	

a See Section 3.3 of ref 15 and Methods of ref 16 for full details.

b See ref 9, in particular their Table 1.

c This extends the effective cutoff to at most twice its nominal value.

d This only applies to the results described in the main section of the manuscript. For the details of the cutoff radius dependent simulations see the Appendix.

### 2.2 Condensed Phase Properties

#### 2.2.1 Thermodynamic Properties

We used *NPT* ensemble simulations (constant number of particles, constant pressure and constant temperature) to compute the heat of vaporization (Δ*H*_vap_), isothermal compressibility (κ), heat capacity (*C*_*p*_) and the coefficient of thermal expansion (α) of six homogeneous liquids (see Section 3.2).^27,28^ Δ*H*_vap_ describes the enthalpy needed to transfer one molecule from a homogeneous, liquid environment into the (ideal) gas phase, i.e., having no interaction with neighbors. The isothermal compressibility κ is a measure of how much a material’s volume decreases under pressure at a constant temperature, and reflects the material’s ability to be compressed without a change in temperature. The heat capacity or thermal capacity *C*_*p*_ is defined as the amount of heat required to raise the temperature of a given quantity of a substance (1 g or mol) by 1 °C (or 1 kelvin). The coefficient of thermal expansion α is a measure of how much a material’s size or volume changes with temperature, and quantifies how much a material expands or contracts when its temperature changes. In classical thermodynamics, κ, *C*_*p*_ and α are defined as $\kappa =-\frac{1}{V}\frac{\partial V}{\partial P},C_{p}=\frac{\partial H}{\partial T},\alpha =\frac{1}{V}\frac{\partial V}{\partial T}$ , where *V* denotes the volume of the system and $\frac{\partial V}{\partial T}$ and $\frac{\partial V}{\partial P}$ denote partial derivatives of *V* with respect to the temperature *T* and the pressure *P*, respectively. *H* denotes the enthalpy of the simulation system. In Supporting Information Section S1, we provide the statistical mechanical derivations how to obtain κ, *C*_*p*_ and α from ensemble averages ⟨.⟩ and fluctuations ⟨Δ.⟩ (variances). These lead to our working equations, eqs 1–4, below; *k*_B_ and *R* denote Boltzmann’s constant and the gas constant.

Δ*H*_vap_ can be obtained from simulations as^27,28^

1 $$\Delta H_{vap}=\left\langle E_{gas}\right\rangle -\left\langle E_{liquid}\right\rangle +RT$$

where *E*_gas_ denotes the energy of a molecule in the gas phase, and *E*_liquid_ denotes the energy of one molecule in the liquid phase. The isothermal compressibility can be obtained as

2 $$\kappa =\frac{1}{k_{B}T}\frac{\left\langle V^{2}\right\rangle -{\left\langle V\right\rangle }^{2}}{\left\langle V\right\rangle }=\frac{1}{k_{B}T}\frac{\left\langle \Delta V^{2}\right\rangle }{\left\langle V\right\rangle }$$

where ⟨*V*⟩ denotes the mean volume of the simulation system, ⟨*V* ^2^⟩ and ⟨*V*⟩^2^ the mean squared volume and the squared mean volume, respectively, and ⟨Δ*V* ^2^⟩ the variance of the volume. The heat capacity is given as

3 $$C_{p}=\frac{1}{NRT^{2}}\left(\left\langle H^{2}\right\rangle -{\left\langle H\right\rangle }^{2}\right)=\frac{1}{NRT^{2}}\left\langle \Delta H^{2}\right\rangle$$

where *N* denotes the number of particles in the simulated system, ⟨*H* ^2^⟩ and ⟨*H*⟩^2^ the mean squared and squared mean enthalpy, respectively, and ⟨Δ*H* ^2^⟩ denotes the fluctuation (variance) of the enthalpy. Finally

4 $$\alpha =\frac{\left\langle VH\right\rangle -\left\langle H\right\rangle \left\langle V\right\rangle }{RT^{2}\left\langle V\right\rangle }=\frac{Cov\left(VH\right)}{RT^{2}\left\langle V\right\rangle }$$

where *T* is the mean temperature and ⟨ Cov­(*VH*)⟩ denotes the covariance of the box volume and the enthalpy.

We also report the average density ρ̂=⟨ρ⟩ . Since the density of a system is proportional to its volume, $\rho \propto \frac{1}{V}$ , the average volume (size of the simulation systems) is implicitly covered by this analysis as well.

#### 2.2.2 Structural and Dynamic Properties

In addition to the physicochemical properties just presented, we also analyzed some structural and dynamic properties. RDFs quantify the average atomic structure in liquids. They describe how, on average, atoms are arranged around each other by placing each atom of the system in turn in the center of a series of concentric spheres, each an interval *dr* larger in diameter than the previous (see Figure 2). The RDF then is computed by

$$g\left(r\right)=\frac{1}{\rho }\frac{1}{4\pi r^{2}dr}\left\langle \frac{1}{N}\sum_{i=1}^{N}\sum_{j\neq i}\delta \left(r-r_{ij}\right)\right\rangle$$

where ρ is the average density for the system, $\frac{1}{4\pi r^{2}dr}$ accounts for the volume of the shell, δ denotes the Dirac delta function, and ⟨.⟩ denotes the mean computed over all frames of the trajectory.

The mean squared displacement (MSD) is a quantitative characterization of diffusion. The MSD describes the process by which atoms located at some position at a given time are found to be in a different position at some time later as a result of random molecular collisions. It is defined as

$$MSD\left(t\right)={\left\langle \frac{1}{N}\sum_{i=1}^{N}|{\mathrm{x⃗}}_{i}\left(0\right)-{\mathrm{x⃗}}_{i}\left(t\right){|}^{2}\right\rangle }_{t}$$

where ${\mathrm{x⃗}}_{i}\left(t\right)$ denotes the Cartesian coordinates of the particle *i* at simulation time *t*. Note that the sum takes all squared displacements $|{\mathrm{x⃗}}_{i}\left(s\right)-{\mathrm{x⃗}}_{i}\left(s+t\right){|}^{2},s\in \left[0,T-t\right]$ into consideration for time lag *t* and simulation length *T*. Using the Einstein-relation, we can use the MSD to compute the self-diffusion coefficient *D* of a liquid as the slope of the MSD combined with a suitable prefactor^41^

$$D=\frac{1}{6}\lim_{t\to \infty }\frac{d}{dt}MSD\left(t\right)$$

## 3 Methods

### 3.1 Choice of NNPs

The decision to focus on just two NNPs, ANI-2x and MACE, was primarily motivated by the availability of a fully trained, transferable model *in addition* to the network implementation itself, together with support in the OpenMM/ML software stack.^32^ Using OpenMM 8.1.1 (see below), all simulations could be carried out immediately, as if a force field had been used.

In parallel with the description of the ANI-2x model,^16^ the trained network was made available and support added to OpenMM.^32^ Recently, the authors of MACE made available a fully trained transferable network for organic chemistry, referred to as MACE-OFF23­(X) and MACE-OFF24­(M), where X = S, M, L (small, medium, and large) denotes the size and complexity of the network.^9^ At the time this study was started, only the MACE-OFF23 models were available. The large version is too slow and memory-intensive for the hardware available to us, so most calculations were carried out with MACE-OFF23­(S), and only a subset of calculations was repeated with MACE-OFF23­(M). All MACE-OFF models are available at https://github.com/ACEsuit/mace-off/; the use of all MACE-OFF models is supported by OpenMM/ML (https://github.com/openmm/openmm-ml/).

These two transferable neural network potentials, ANI-2x and MACE-OFF­(S/M) embody different approaches for enforcing the symmetry and locality constraints crucial in modeling molecular interactions. In particular, the models not only differ in their network architectures, but also in how they construct the representation of a molecular system. One potentially relevant practical difference between the networks is their range. ANI-2x operates with a strict, short cutoff radius (4.6 Å for the radial and 3.1 Å for the angular symmetry functions^15^). The primary cutoff radius of MACE-OFF is similarly short (4.5 Å/5 Å/5 Å for S/M/L, respectively^9^); however, because of message passing, the effective range is extended, allowing information flow from the entire cutoff sphere of each neighbor j∈N(i) to the central atom *i* (see Figure 1).

OpenMM-ML also supports another trained family of NNPs called Nutmeg,^42^ which uses the TensorNet architecture,^43^ trained on the SPICE 2 data set.^42^ However, as stressed by the authors themselves, this NNP family does not work well for liquid systems. We did reproduce the O–O RDF for bulk water, shown in Figure 9 of ref 42, and observed the formation of large, persistent cavities, thus confirming the authors’ warning. Therefore, Nutmeg potentials were not included in the analysis presented in this work.

### 3.2 Choice of Systems

We carried out *NPT* and *NVT* (constant number of particles, constant volume and constant temperature) simulations of six homogeneous liquids: water, methanol, acetone, benzene, and *n*-hexane at room temperature, as well as NMA at 373.15 K (see Figure 3). Given its essential role in biological systems, the detailed look at water properties needs no further justification. Methanol was chosen as the most “water-like” organic solvent. Acetone represents a polar organic solvent, whereas benzene and *n*-hexane are representative examples of aromatic and aliphatic apolar solvents, respectively. In classical force field development, it has been and is considered essential to have good parameters for NMA as a model compound for the peptide bond.^29^ Hence, it seems relevant to look at how well NNPs reproduce the condensed phase properties of liquid NMA. Since literature data for NMA are not sufficiently accurate at lower temperatures,^44^ all major force fields carry out NMA based parametrizations at 373 K.^28–30^ Therefore, our NMA simulations were carried out at 373.15 K.

**Figure 3:**
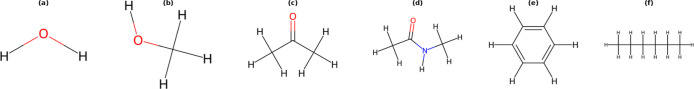
Species in homogeneous simulation systems. (a) Water. (b) Methanol. (c) Acetone. (d) *N*-Methylacetamide (NMA). (e) Benzene. (f) *n*-Hexane.

### 3.3 Simulation Details

The simulation systems were built using CHARMM-GUI.^45–47^ The box sizes were chosen as small as possible to avoid extensive computation times in the NNP simulations, but large enough so that the box length *L*/2 > *r*_c_ = 12 Å, the cutoff radius *r*_c_ appropriate for the CHARMM force field family (see below). All details about the systems and box sizes are listed in Table 2.

**Table 2:** Simulation Details^a^

	water	methanol	acetone	benzene	*n*-hexane	NMA
# molecules	572	323	178	130	98	146
# atoms	1716	1938	1780	1560	1960	1764
*L*_*NPT*,initial_ [Å]	25.67	27.99	27.95	27.21	27.72	26.97
*L*_*NVT*_ [Å]	25.80	27.98	27.99	26.72	27.77	NA

a *L* denotes the length of the simulation boxes, i.e. *V* = *L* ^3^. *L*_*NPT*,initial_ is the box length after 10 ns force field equilibration. *L*_*NVT*_ corresponds to the value appropriate for the experimental density.

All simulations were carried out with OpenMM 8.1.1.^32^ In the force field baseline calculations, we employed CGenFF for the organic molecules;^34–36^ water was modeled as TIP3P.^33^ Lennard-Jones interactions were switched off between 10 and 12 Å; electrostatic interactions were calculated by particle-mesh-Ewald (PME) summation.^40^ The OpenMM/ML functionality^32^ was used for the NNP simulations. Specifically, torchani^48^ was employed for the ANI-2x and mace-torch (PyTorch implementation of MACE, available on https://github.com/ACEsuit/mace) for the MACE-OFF23 simulations. To investigate the dependence of water properties on the cutoff radius, we carried out a series of additional force field runs using CHARMM^49^ (see the Appendix).

Except for the TIP3P simulations, all molecules were fully flexible, so we used an integration time step Δ*t* = 0.5 fs. We carried out simulations in the *NPT* and *NVT* ensembles. In the *NPT* simulations, we used Langevin dynamics with a friction coefficient of 1/ps to control the system’s temperature and the Monte Carlo barostat^50,51^ to maintain an average pressure of 1 atm. To compute the heat of vaporization, we ran simulations in the gas phase by simulating a single molecule without periodic boundary conditions. The required energy in the gas phase was computed as the mean value over the gas phase trajectory. To calculate MSDs and self-diffusion coefficients, we also carried out *NVT* simulations using a Nosé–Hoover thermostat.^52^ The average temperature of the water, methanol, acetone, benzene and *n*-hexane simulations was held around 300 K. The NMA simulations were carried out at 373.15 K. To ensure comparability across all methods (NNPs and force field) for each of the six liquids studied, we set the box volume in the *NVT* simulations to match the respective *experimental* density. We note already here that in some cases this choice of box volume led to sizable discrepancies from the densities to which the systems converge in *NPT* simulations (see Results and Discussion).

The workflow/order in which simulations were carried out is summarized in Figure 4. Each of the systems was first simulated for 10 ns at the force field level, both under *NPT* and *NVT* conditions. Then, we carried out 100 ps of equilibration at the respective level of theory (MM, ANI-2x, MACE), followed by a 1 ns/3.5 ns production simulation under *NPT* and *NVT* conditions, respectively. As we observed slow convergence in some cases (see Results and Discussion), we used the final configuration of each *NPT* simulation to start five additional, independent *NPT* simulations, assigning new random velocities. In each run, 100 ps were discarded as (re)­equilibration, followed by 1 ns of production. This full series of simulations as just described (*NPT* production simulations of 1 + 5 × 1 ns, one *NVT* 3.5 ns simulation) was carried out for MM, ANI-2x and MACE-OFF23­(S).

**Figure 4:**
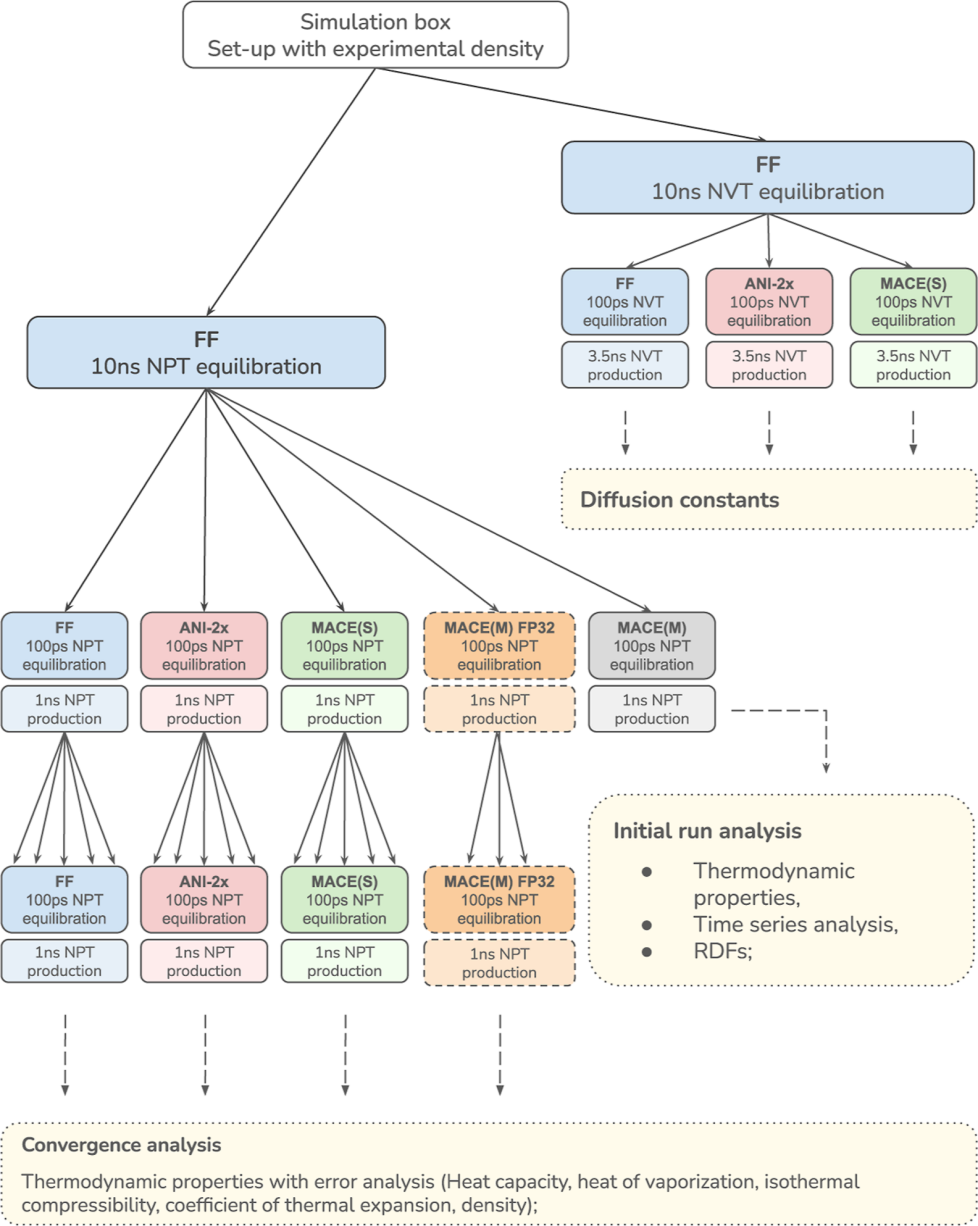
Overview of all simulations carried out. The dashed lines around the boxes for the MACE-OFF23­(M) model in FP32 floating-point arithmetic indicate that this set of simulations, consisting of one initial *NPT* run and three independent repetitions, was only done for a subset of systems (water, acetone, *n*-hexane).

All simulations were carried out on Nvidia RTX 4090 and RTX6000 Ada GPUs. Most NNP energy and force calculations used double precision floating-point arithmetic (FP64), as in the MACE-OFF study;^9^ see also ref 53. Since on our computer resources MACE-OFF23­(M) in FP64 was extremely slow (see Supporting Information, Tables S14 and S15, and Conclusions), we did not carry out *NVT* simulations and only performed one 1 ns *NPT* simulation for this model. To gain some insights into the performance of the medium model, we performed a subset of simulations with MACE-OFF23­(M) in single precision floating-point arithmetic (FP32). These additional FP32 calculations were carried out only for water, acetone, and *n*-hexane, with 3 instead of 5 repeats after the initial 1 ns simulation, as indicated in Figure 4.

### 3.4 Data Analysis

The quantities required to compute the physicochemical properties of interest, e.g., instantaneous volume and potential/total energy, were logged every 100 steps during the *NPT* simulations and used to estimate the required ensemble averages and fluctuations (see eqs 1–4).

To monitor convergence, particularly during the initial 1 ns simulation, we also computed time-series for Δ*H*_vap_, κ, *C*_*p*_, α, and ρ by replacing ensemble averages ⟨.⟩ in eqs 1–4 and ρ by ⟨.⟩_*t*_, where ${\left\langle X\right\rangle }_{t}≔\frac{1}{t}\sum_{i=0}^{t}X_{i}$ denotes the statistical mean over all data points in the trajectory up to time point *t*. These analyses indicated that convergence may not be achieved within a single nanosecond, and prompted the additional five repeats for each system (cf. above). In the results below, no uncertainty estimates are reported for any properties derived from the initial production runs of 1 ns length. For the five repeated simulations, statistical errors were estimated by the standard deviation between the five independent 1 ns simulations (cf. above) as the more reliable uncertainty estimate (cf. Supporting Information Tables S7–S12).

We also saved coordinates to disk every 100 steps; RDFs and MSDs were calculated from these trajectories. RDFs for selected atom–atom pairs were computed using the MDAnalysis package.^54,55^ The RDFs shown in Results and Discussion were computed using the entire trajectory from the initial 1 ns *NPT* simulation runs (cf. above). RDFs obtained from the additional repetitions were indistinguishable in all cases. The MSDs (and diffusion coefficients) based on molecular centers of mass were calculated with the NewAnalysis package developed in-house^56^ using the trajectories saved during the *NVT* simulations.

## 4 Results and Discussion

### 4.1 Water: First Results and Convergence

Before presenting the thermodynamic/physicochemical properties of all six liquids studied, we take a look at the results for water obtained from the first 1 ns production simulation. Our results are depicted in Figure 5. Here and in all remaining plots of this manuscript, force field (FF) results are shown in blue, ANI-2x in red, MACE-OFF23­(S) in green, and MACE-OFF23­(M) in gray. The detailed data obtained during the initial 1 ns simulation for water, as well as the other five liquids, can be found in Supporting Information, Tables S1–S6. Figure 5a shows time-series of selected thermodynamic properties, from top to bottom: heat capacity *C*_*p*_, isothermal compressibility κ, and density of water ρ. No data are shown for the heat of vaporization as it converges rapidly, given that it is computed as a mean value over all molecules in the simulation system. One sees immediately that despite an equilibration of 100 ps (cf. Methods), the ANI-2x results vary considerably during the 1 ns simulation, i.e., the ANI-2x results are unlikely to have converged. For *C*_*p*_ and κ, this is obvious, but also ρ (bottom plot) shows a drift. The small, continuous decline of the ANI-2x density from a slightly too high starting value is partially obscured, since both MACE models result in densities that are significantly too high. The plots in Figure 5a show that for water described by ANI-2x 100 ps of equilibration, followed by 1 ns of production, are not sufficient to obtain converged results. Similarly slow, or even insufficient convergence was observed for some other liquids and models, which prompted the additional 5 × 1 ns productions simulations using FF, ANI-2x, and MACE-OFF23­(S); cf. Methods.

**Figure 5:**
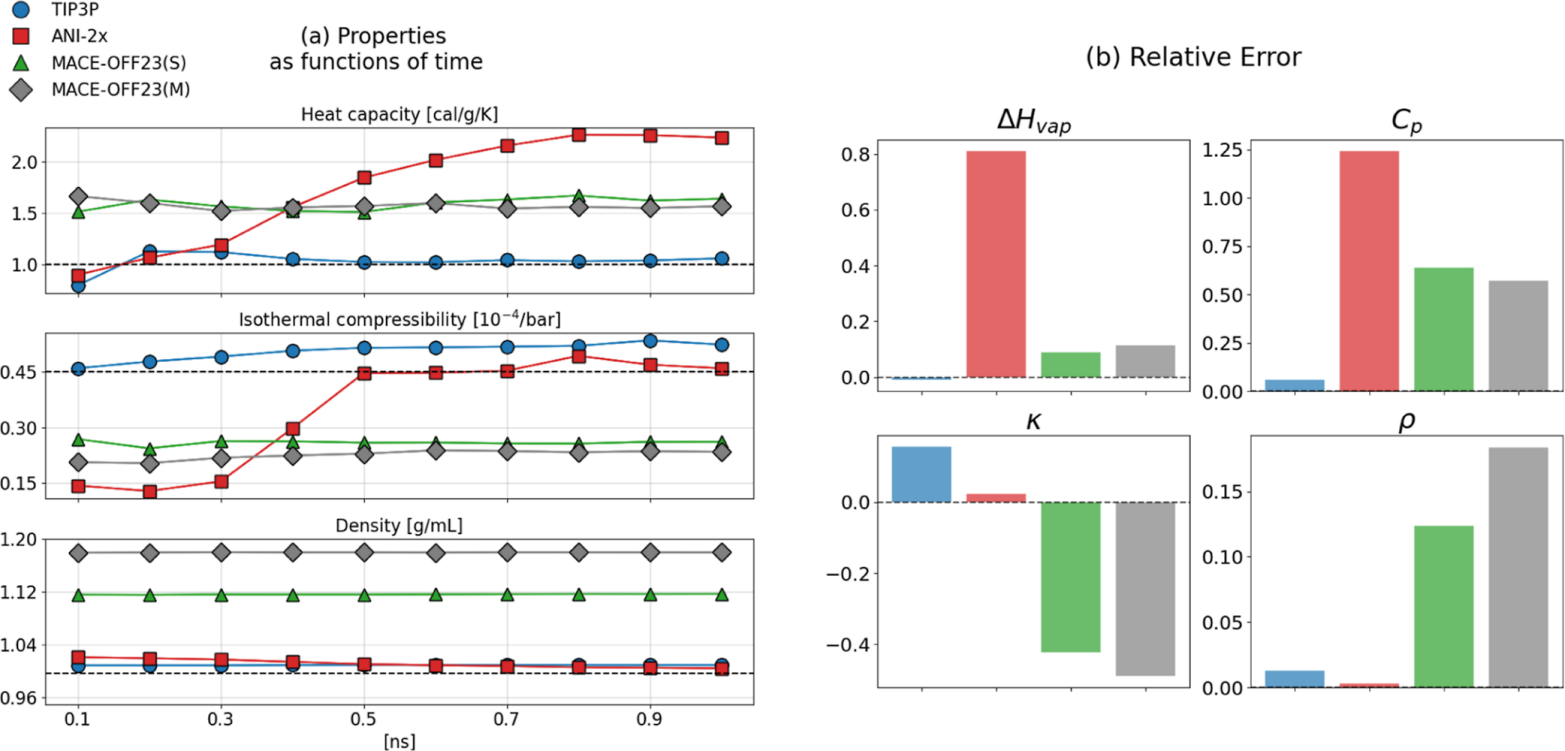
(a) Time-series for (from top to bottom) heat capacity *C*_*p*_, isothermal compressibility κ, and density ρ of water. For each plotted data point, an additional trajectory subpart of length 100 ps was included in the computation of the average. That is, the first data point was computed from the initial 100 ps of the production run trajectory and the final data point was computed from the entire 1 ns production run data. In each subplot of (a), the dashed horizontal line denotes the experimental value; (b) relative errors (eq 5) for (from left to right, top to bottom) heat of vaporization Δ*H*_vap_, heat capacity *C*_*p*_, isothermal compressibility κ, and density ρ for 1 ns simulation runs for water using the TIP3P water model (blue), ANI-2x (red), MACE-OFF23­(S) (green) and MACE-OFF23­(M) (gray).

In Figure 5b, we show the relative error

5 $$\epsilon_{rel}=\frac{\mathrm{x̂}-x_{\exp}}{x_{\exp}}$$

for all water results (force field, ANI-2x, MACE-OFF23­(S) and MACE-OFF23­(M)) obtained during the initial 1 ns simulation. In eq 5, *x̂* is the computed estimate for Δ*H*_vap_, *C*_*p*_, κ, and ρ, and *x*_exp_ denotes the respective experimental value. One observes immediately that ANI-2x massively overestimates the heat of vaporization, and even more so *C*_*p*_. Both MACE-OFF23­(S) and MACE-OFF23­(M) have a low error for Δ*H*_vap_, but also overestimate *C*_*p*_. The two MACE models predict densities that are too high and isothermal compressibilities that are too low. For these two quantities, ANI-2x is in the best agreement with experiment. However, given ANI’s poor performance for Δ*H*_vap_ and *C*_*p*_, it is fair to conclude that, overall, the TIP3P water model^33^ is in the best agreement with the experimental data.

### 4.2 Thermodynamic Properties

In Figure 6 we summarize the results for all computed properties (Δ*H*_vap_, *C*_*p*_, κ, α, and ρ) and all six systems studied (water, methanol, acetone, NMA, benzene, and *n*-hexane). The small solid symbols indicate the mean value of the five 1 ns simulations, restarted from the final configuration of the initial 1 ns production calculation (cf. Methods). The respective standard deviation *s* is indicated as whiskers; when *s* is small, these are barely discernible. The results from the initial 1 ns simulations (see Supporting Information, Tables S1–S6) are displayed as slightly larger, transparent symbols. All raw data from the repeated 1 ns simulations are tabulated in Supporting Information, Tables S7–S12. As described in Methods, for MACE-OFF23­(M) we carried out only a single 1 ns simulation in FP64. The subset of MACE-OFF23­(M) FP32 results is shown in orange in Figure 6. The experimental values (where available) are indicated as a dashed black line. Off-scale results are indicated by arrows.

**Figure 6:**
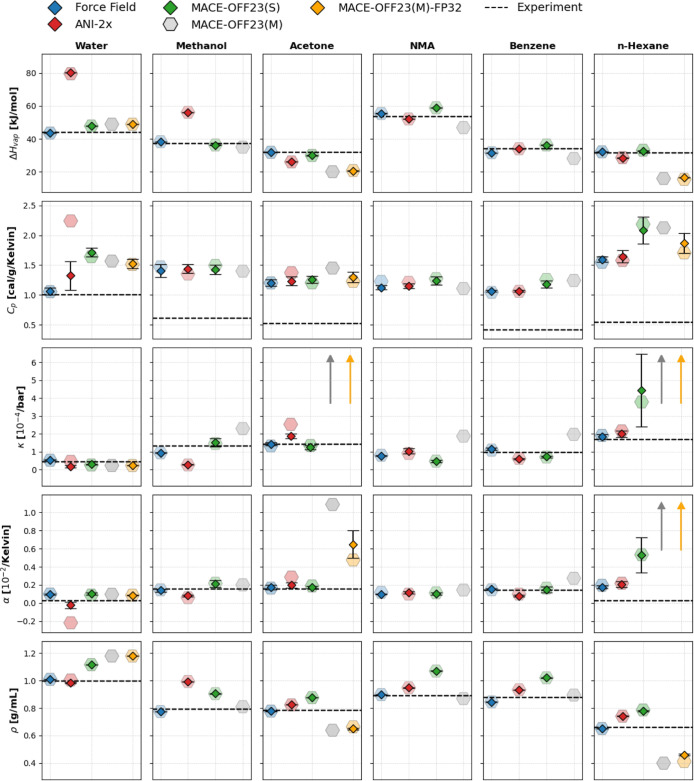
Summary of physicochemical and thermodynamic properties for all investigated systems. The larger, transparent symbols are the results for the initial *NPT* production simulation (FF, ANI-2x, MACE-OFF23­(S) and MACE-OFF23­(M)). The smaller symbols with whiskers indicate the mean ± standard deviation of five independent runs (FF, ANI-2x and MACE-OFF23­(S)). The subset of MACE-OFF23­(M) results in FP32 precision is shown in orange. The horizontal dashed line denotes the experimental value in each subplot, if available. Experimental heat of vaporization values, density of NMA and all other experimental values were taken from refs 57–59 , respectively.

The water results in Figure 6 further highlight the convergence problems already discussed in the previous section. E.g., the ANI-2x water results (red symbols) for *C*_*p*_, α, and κ clearly are different between the initial 1 ns and the mean value of the subsequent five 1 ns simulations (see also Tables S1 and S7). The *C*_*p*_ value estimated from the five repetitions is in significantly better agreement with the experiment, but the standard deviation remains high. The mean value from five simulations for the coefficient of thermal expansion of ANI-2x water shows a noticeable drift compared to the initial run, and the agreement with experiment improves. On the other hand, the computed isothermal compressibility of ANI-2x water obtained as the mean value of five repetitions is in poorer agreement with experiment compared to the value estimated based on the first 1 ns simulation.

The ANI-2x results for acetone also show indications of slow convergence (i.e., results from the first nanosecond and the mean of the five 1 ns simulations are different). Most MACE-OFF23­(S) results (green symbols) look well converged, except for the hexane results. Here, clear differences between the initial 1 ns and the mean of the five 1 ns results are discernible, and the standard deviation of the latter is high. Given the complexity of sampling the internal conformations of hexane, even five 1 ns simulations may be insufficient, but neither the force field (blue), nor ANI-2x (red) exhibits this behavior. In several cases, the MACE-OFF23­(M) model has the largest deviation from experiment and the other methods (FF, ANI-2x, and MACE-OFF23­(S)). First, the density of water is even higher than that of MACE-OFF23­(S); see the previous section and ref 9. By contrast, the density of acetone and hexane is considerably too low. Reference 9 does not list the densities of the studied systems in numerical form, but their Figure 5 shows that the medium model significantly underestimates the density of several liquids, especially when the experimental density is lower than that of water. The too low density is most likely responsible for the too high compressibility κ, which for hexane leads to values that are off-scale, as indicated by the arrows. We visually inspected the MACE-OFF23­(M) FP64 trajectory of hexane and observed the intermediate formation of cavities (see Supporting Information Figure S1). Thus, the MACE-OFF23­(M) simulations for hexane are certainly not converged, and it is arguable whether they can be considered stable. As an aside, the FP64 (gray) and FP32 results (orange) for water agree well, whereas there are noticeable differences for acetone and, in particular, hexane.

In Figure 7, to facilitate the comparison to experiment, we show correlation plots between computed and experimental data for the heat of vaporization, density, and heat capacity; the theoretical *y* = *x* line is included as visual guidance (dashed, black line). In the figure, we only distinguish between models, using our usual color code (FF blue, ANI-2x red, MACE-OFF23­(S) green, MACE-OFF23­(M) gray). Individual data points can be identified by referring either to Figure 6 or Supporting Information, Tables S7–S12. Since we could not find experimental heat capacity data for NMA under our simulation conditions, NMA results are not included in Figure 7c. For MM, ANI-2x and MACE-OFF23­(S) we plot the mean values of the five 1 ns simulation results. For MACE-OFF23­(M), the result obtained from the single 1 ns simulation carried out is included.

**Figure 7:**
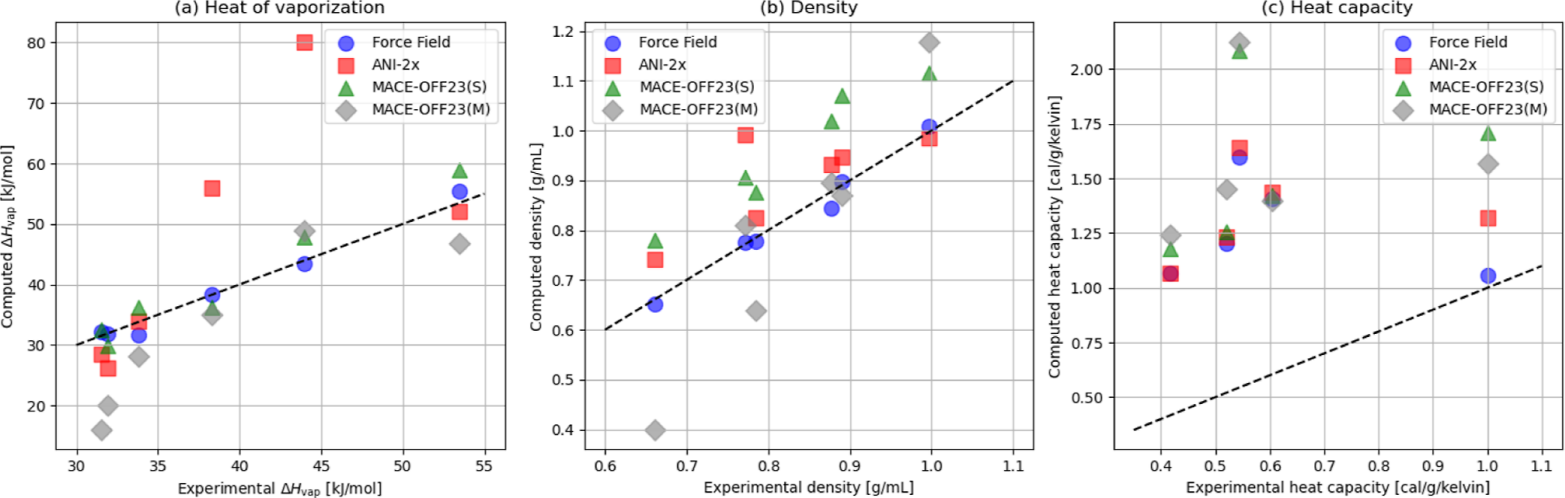
(a) Heat of vaporization, (b) density and (c) heat capacity compared to the respective experimental values. The dashed line in each of the subplots denotes the linear function *y* = *x*. Since no experimental heat capacity data are available for the simulation conditions, the NMA *C*_*p*_ is not included in subplot (c).

Figure 7a shows that ANI-2x in two cases leads to heats of vaporization in poor agreement with experiment. These two outliers in Figure 7a are methanol and water—small molecules containing an OH group. The ANI-2x heats of vaporization for the other four systems are in good, or at least acceptable, agreement with experiment. In contrast, the force field generally yields heats of vaporization that closely match experimental measurements. The Δ*H*_vap_ results obtained with MACE-OFF23­(S) are also in good agreement with the experiment, whereas the MACE-OFF23­(M) results for acetone and hexane deviate significantly.

The force field also performs well for the density (Figure 7b). The ANI-2x results are acceptable, with one exception (methanol), where the calculated density is significantly overestimated. All MACE-OFF23­(S) densities are too high, as reported in ref 9. The MACE-OFF23­(M) densities are inconsistent, some are in acceptable agreement with experiment, but some are significantly too high (water) or too low (acetone, hexane). All *C*_*p*_ results (Figure 7c) are in poor agreement with the experiment, with the single exception of the force field result for water (see also Figure 5).

To put these findings into perspective, one should remember that force fields are highly optimized against properties such as the heat of vaporization and density. Consequently, the good agreement with these properties is expected rather than surprising. Their calculation using NNPs is an application far outside their training domain. The ANI-2x developers certainly never trained their model against condensed phase properties; the MACE-OFF publication^9^ reports only Δ*H*_vap_ and ρ.

### 4.3 Radial Distribution Functions

In Figure 8, we show a selection of RDFs, one for each liquid. For water (Figure 8a), we compare the oxygen–oxygen RDF for ANI-2x (red), MACE-OFF23­(S) (green), MACE-OFF23­(M) (gray, dotted), and the CHARMM force field (TIP3P, blue). We also include experimental data for the O–O RDF (black, dashed curve).^60^ One sees immediately that the ANI-2x RDF has significantly too much structure, in particular, the first minimum and second maximum are too pronounced. Possible reasons for the too much pronounced structure in ANI-2x water, which has been observed by others as well,^9,24^ is investigated further in the Appendix, where we explore the effect of cutoff on the RDF of TIP3P.

**Figure 8:**
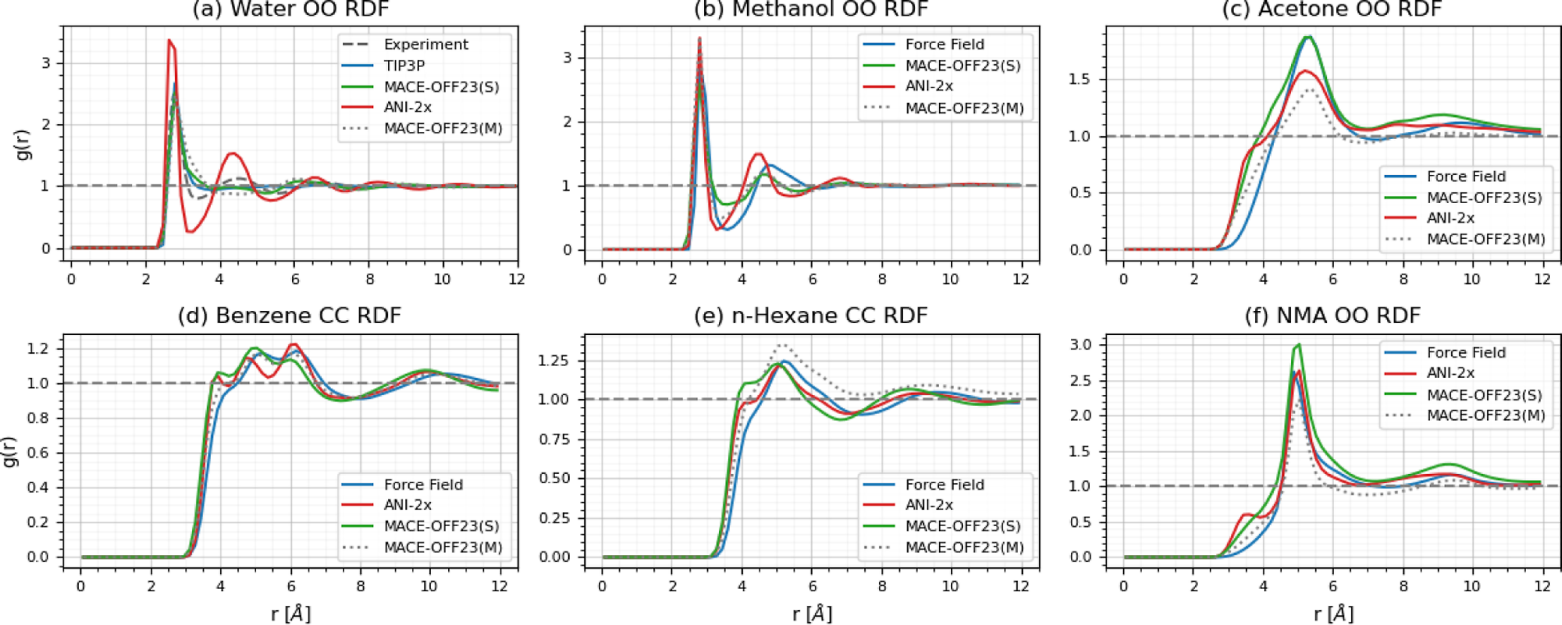
Selected radial distribution functions: (a) water oxygen–oxygen RDF. (b) Methanol oxygen–oxygen RDF. (c) Acetone oxygen–oxygen RDF. (d) Benzene carbon–carbon RDF. (e) *n*-Hexane carbon–carbon RDF. (f) NMA oxygen–oxygen RDF. Each plot contains a radial distribution function computed from an *NPT* ensemble simulation using ANI-2x, MACE-OFF23­(S) and MACE-OFF23­(M), as well as an RDF computed from a force field simulation. The water oxygen–oxygen plot additionally includes the experimental RDF.^60^

The MACE-OFF23­(S) RDF is shaped similarly to experimental data; however, the second maximum is too low and, in fact, remains below 1. Overall, it exhibits less structure than the TIP3P RDF (blue). Using the MACE-OFF23­(M) model, the first minimum of the oxygen–oxygen RDF is shifted noticeably to the right. The shallow first minimum is centered at the position of the experimental second maximum. Since the *NPT* simulations using MACE resulted in densities that deviated significantly from the experimental value, we also compared water RDFs obtained from the *NPT* and the *NVT* simulations. In the latter (cf. Methods), the box size was set to reproduce the experimental water density. For water described by MACE-OFF23­(S), the RDFs obtained from *NPT* and *NVT* simulations are shown in Supporting Information Figure S2. Use of the *NVT* ensemble (black)—with the “forced” correct experimental density—leads RDFs in reasonable agreement with experiment (gray). By contrast, in the RDF computed from the *NPT* simulations (green)—with a density that is ≈10% too high—the first minimum and second maximum are less pronounced and, as already mentioned, the second maximum does not exceed the value of 1. The marked difference between RDFs obtained from *NPT* and *NVT* simulations indicates that large density errors also influence local structure.

Similarly to water, ANI-2x also results in “more” structure for the O–O RDF of methanol (Figure 8b) compared to the other methods (FF and MACE). The second maximum of the ANI-2x RDF is significantly more pronounced; furthermore, the first minimum and second maximum are shifted to the left. Given that both water and methanol contain an OH-group, this is not totally surprising. In the remaining RDFs shown, ANI-2x always results in the most accentuated curves. E.g., the individual maxima and minima in the carbon–carbon RDF for benzene are most pronounced for ANI-2x, and only the ANI-2x RDF has a pronounced shoulder at ≈3.5 Å for acetone. Similarly, the ANI-2x oxygen–oxygen RDF for NMA has an additional maximum at short distances. The RDFs obtained with the FF (blue) and the two MACE models (green and gray) also show clear differences, which lacking experimental data cannot be interpreted. Nevertheless, the plots clearly show that local structure as reflected by RDFs is noticeably different when using the small and medium MACE models, respectively; this is particularly visible for acetone and hexane.

### 4.4 Self-Diffusion Coefficients

Figure 9 shows the logarithm of the center-of-mass self-diffusion coefficients for water, methanol, acetone, benzene, and *n*-hexane (cf. Methods). All numeric values used to create the plot are listed in Supporting Information, Table S13. One immediately notices that ANI-2x underestimates water diffusion, yielding a value that is 3 orders of magnitude slower than the experimental value. In contrast, TIP3P is known to slightly overestimate the self-diffusion coefficient of water,^62^ and MACE-OFF23­(S) accurately reproduces the experimental value.

**Figure 9:**
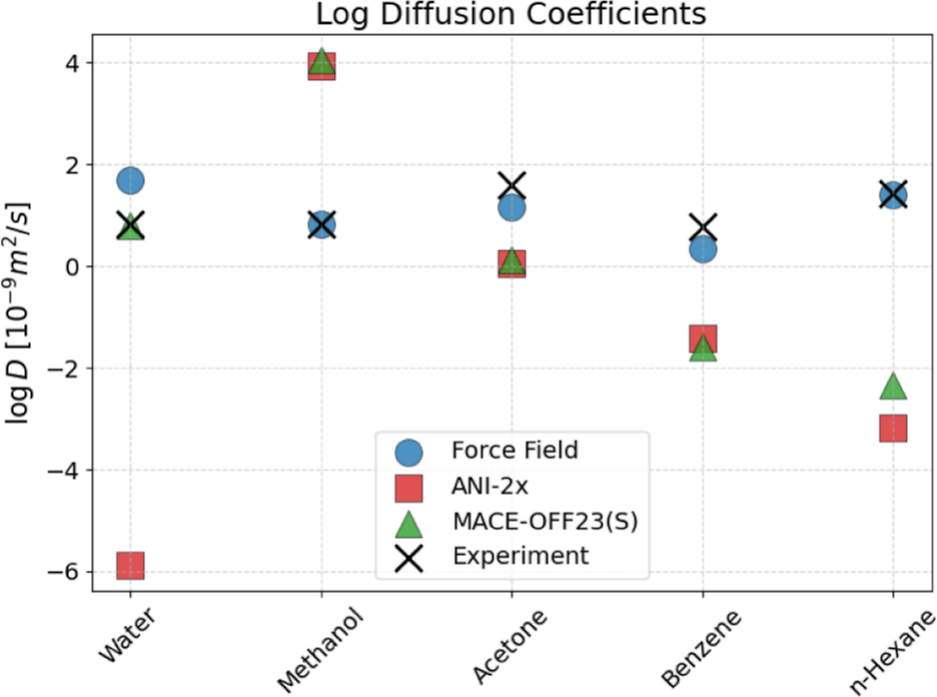
Logarithmic self-diffusion coefficients. The experimental values are taken from ref 61.

The force field consistently leads to self-diffusion coefficients that are within the same order of magnitude as the experimental values. For methanol, both ANI-2x and MACE-OFF23­(S) result in too fast diffusion. By contrast, both NNPs result in too low diffusion constants for acetone, and, in particular, benzene and hexane. In the Appendix, similarly to the water O–O RDF, we present data for the TIP3P diffusion constant as a function of cutoff radius in an attempt to understand the particularly poor and surprising ANI-2x result for water.

## 5 Concluding Discussion

### 5.1 Summary

We already stated that in terms of thermodynamic/physicochemical properties, the force field performed by far the best, see Figures 6 and 7. The comparison of RDFs to experimental data is difficult and possible only for water (Figure 8a). As discussed, the ANI-2x model results in far too much structure, and the MACE-OFF23­(M) model results in a water O–O RDF where the first minimum is located at the position of the second maximum found in experiment. Only the force field simulations gave self-diffusion coefficients that had the correct order of magnitude for the five liquids for which experimental data are available. By contrast, both ANI-2x and MACE-OFF23­(S) resulted in sizable errors (more than 1 order of magnitude) for at least one system (Figure 9).

We investigated possible reasons for the severe artifacts observed for ANI-2x water (see the Appendix). If TIP3P water is simulated with extremely short cutoff radii (≤5 Å), it starts to behave similarly to ANI-2x. These findings suggest that too short cutoffs without message passing should be avoided. While the primary cutoff radius of MACE is comparable to that of ANI-2x, the effective cutoff is enlarged because of message passing.

While not immediately related to the quality of the results, at present using NNPs to study liquids is quite costly (see Supporting Information, Tables S14 and S15). Even ANI-2x is almost 300 times slower compared to the force field calculations. MACE-OFF23­(S) is a factor of 6 slower than ANI-2x, and the medium model in FP64 achieves less than 50 ps/day. To achieve any reasonable performance in double precision requires the use of the costly highest-end NVIDIA cards (A100, H100 or better) since even on the professional RTX 6000 Ada models the FP64 performance is crippled. In addition, the memory requirement for the MACE-OFF23­(M) model in FP64 precision is also quite high (>40 GB GPU RAM for the system sizes studied here).

### 5.2 Consequences of (Too) Slow Convergence

Arguably, the most striking failure observed in this study is the poor representation of water properties when using ANI-2x. The heat of vaporization is far too high, the radial distribution functions show “too much” structure, and the self-diffusion constant is too low by 3 orders of magnitude. The significantly reduced mobility of individual water molecules is reflected by the slow convergence of properties when using ANI-2x; cf. Section 4.1. It is tempting to discard these findings as artifacts relevant to ANI-2x and boxes of water, but we argue that these shortcomings make ANI-2x unsuitable for any simulations involving water.

In molecular dynamics simulations, the expectation value ⟨X⟩ of some quantity of interest, *X*, can be estimated by following the time evolution of the system (Boltzmann view)

6 $$\left\langle X\right\rangle =\lim_{T\to \infty }\frac{1}{T}\int_{0}^{T}X\left(t\right)dt$$

According to the Ergodic hypothesis, both approaches (Boltzmann averaging and the theoretical representation of the Boltzmann distribution) should yield equivalent results. MD simulations mimic the Boltzmann approach; however, one obviously can only average over finite time intervals *T*, which will only give an approximate of the expectation value

7 $$\left\langle X\right\rangle \approx \frac{1}{T}\int_{0}^{T}X\left(t\right)dt,,T\in R_{+}$$

Thus, if the dynamics of (parts of) a system is slowed down, then estimating the expectation value will converge slowly as well. E.g., when calculating the average internal energy of our water box over 5 repeats of 1 ns each, the standard deviation of the five averages was considerably higher using ANI-2x compared to FF or MACE.

Ensemble averages ⟨X⟩ are central to many applications of MD simulations, including the calculation of free energy differences.^63^ If the dynamics of a system (or the solvent part of a system) is as dramatically distorted as with ANI-2x, then convergence will not occur during typical simulation lengths of one or a few nanoseconds. This would be bad enough using force fields; when using NNPs that are several hundred times slower to compute, the artificially slow convergence is prohibitive.

### 5.3 Outlook

The need to validate NNPs beyond the reproduction of energies and forces is being recognized,^9,23,25,26^ and some issues reported here have been observed by others. E.g., the authors of MACE-OFF^9^ acknowledge that the small model tends to overestimate densities. However, for the six liquids scrutinized in this study, the medium model (MACE-OFF(23)) does not necessarily improve the results. Arguably, the most worrisome conclusion of this work is that shortcomings of a model can make it completely unsuitable for seemingly unrelated applications. Specifically, the far too slow self-diffusion of ANI-2x water renders simulations of any solute in this environment useless, as exceedingly long simulations would be required for convergence.

The developers of classical force fields have recognized the importance of condensed phase properties, such as the heat of vaporization, for a long time.^27–30^ How to incorporate the reproduction of condensed phase properties in the development of transferable NNPs, however, is currently unclear. The so-called fourth generation NNPs are augmented with long-range corrections, e.g., ref 64; this may be a hook to target and improve condensed phase properties. In this context, it should be noted that water properties obtained from ab initio MD simulations are highly dependent on the DFT functional used.^65,66^ Improvements can be obtained by adding dispersion corrections.^67–69^ A recent study demonstrated that using the more costly, yet more accurate Minnesota functionals resulted in much better agreement between simulated and experimental water properties, thus eliminating the need for dispersion corrections.^70^

NNPs may work well in many applications, but our cursory inspection of six liquids strongly suggests that one should exercise care when applying the current generation of transferable models to study condensed phase systems.

## Supplementary Material



## Figures and Tables

**Figure 10:**
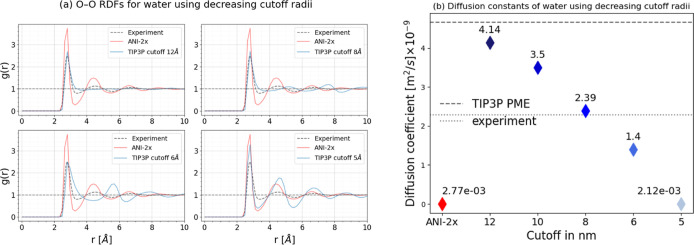
(a) Oxygen–oxygen radial distribution functions of TIP3P water computed with decreasing cutoff radii *r*_c_ ∈ {12, 8, 6, 5} Å. (b) Self-diffusion coefficients for TIP3P water simulations as a function of cutoff radius.

## Data Availability

A collection of the scripts that were used to compute the here presented results, as well some exemplary data reports and all computed properties can be found in the github repository https://github.com/cbc-univie/cp_properties.

## Appendix

### The Possible Role of Cutoff for ANI-2x’ Water Properties

To explore the origin of the over-structured ANI-2x RDF as well as the too low self-diffusion coefficient of water, we compared the ANI-2x results to those obtained by force field simulations with shorter and shorter cutoff radii *r*_c_. The TIP3P simulations were carried out with CHARMM^49^ using a simulation box containing 1000 water molecules. For each *r*_c_, we carried out 5,000,000 MD steps with a 2 fs integration step (i.e., a simulation length of 10 ns). In all simulations, we applied CHARMM’s potential *shifting* to Coulomb and Lennard-Jones interactions^71^ to mimic the cutoff/tapering function implemented by the ANI-2x model.

Figure 10a displays RDFs for TIP3P water as a function of cutoff radius. Each subplot shows the RDF from the TIP3P simulation (blue), indicating the cutoff, the ANI-2x OO RDF (red), and the experimental OO RDF (black, dashed curve). For a 12 Å cutoff, the RDF for TIP3P shows little structure beyond the first shell, as expected for TIP3P. As *r*_c_ decreases, and, in particular, for *r*_c_ = 5 Å, the RDF exhibits noticeably more structure, resembling the behavior of ANI-2x. We also calculated the diffusion constant of the TIP3P simulations as a function of the cutoff radius; the results are displayed in Figure 10b. As the cutoff radius becomes shorter, the self-diffusion coefficient of TIP3P water slows down. For *r*_c_ = 5 Å, the diffusion constant drops by three orders of magnitude, which is close to the ANI-2x value.

Thus, we can reproduce the ANI-2x water properties by force field simulations with a cutoff that is similar to that of the ANI-2x model. This suggests that the use of a very near-sighted NNP *without* message passing (as is used in the MACE and most other NNPs) may cause severe artifacts for condensed phase properties. Water, as a small, highly polar molecule, is particularly affected since a cut-off radius of ≤5 Å excludes any interactions beyond nearest neighbors.

## Notes

(i) The detailed mathematical derivations of thermodynamic properties, (ii) additional plots, and (iii) tables listing all numeric values presented in this work (PDF)
