# Supplementary material for: Transferable Neural Network Potentials and Condensed Phase Properties
Source: J Chem Inf Model. 2025 Sep 11;65(18):9483–96. doi: 10.1021/acs.jcim.5c01355 (PMC12458686; doi:10.1021/acs.jcim.5c01355)
Supplement: Supplementary file 1 [file ci5c01355_si_001.pdf]

Supplementary Information – Transferable neural network potentials and condensed phase properties

Anna Katharina Picha,[†,¶] Marcus Wieder,[‡] and Stefan Boresch[*,†]

†*University of Vienna, Faculty of Chemistry, Institute of Computational Biological Chemistry, Austria*

‡*Open Molecular Software Foundation, Davis, California 95616, United States*

¶*University of Vienna, Vienna Doctoral School of Chemistry (DosChem), Austria*

E-mail: stefan.boresch@univie.ac.at

1 Derivation of thermodynamic quantities from statistical mechanics

Since it is difficult to find the derivation of thermodynamic properties in terms of ensemble averages in textbooks, we present it here for the reader's convenience. The goal is to derive statistical formulas for each of these properties from their thermodynamic definitions. To achieve this, we use the following representation of the Gibbs free energy G:

$$G = -k_B T \ln(Z), \tag{1}$$

where Z is the partition function in the NPT ensemble:

$$Z = \frac{1}{N!}\frac{1}{h^{3N}} \int\limits_{\mathbb{R}^{3N}} \int\limits_{\mathbb{R}^{3N}} \int\limits_{\mathbb{R}_+} e^{-\beta[\mathcal{H}(q,p)+PV]} dq\; dp\; dV. \tag{2}$$

Here, $\mathcal{H}(q,p)$ is the Hamiltonian as a function of coordinates q and momenta p, h denotes the Planck constant and $\beta = \frac{1}{k_B T}$. P and V denote pressure and volume. The Cartesian coordinates q and momenta p both are elements of the 3-dimensional, real-valued space $\mathbb{R}^{3N}$ and the volume V is a (positive), real-valued number, i.e. $V \in \mathbb{R}_+$. For convenience, the integration domains $\mathbb{R}^{3N}$, $\mathbb{R}^{3N}$ and $\mathbb{R}_+$ will be omitted in notation for the following parts. In general, expectation values can be computed by

$$\langle X \rangle = \int X(r) f(r) dr, \tag{3}$$

for some density function $f(r)$. Here, in the NPT ensemble, we consider ensemble averages as expectation values that are computed from a Boltzmann distribution with density function $f(q,p,V)$ where

$$\begin{aligned} f(q,p,V) &= \frac{e^{-\beta[\mathcal{H}(q,p)+PV]}}{\int\int\int e^{-\beta[\mathcal{H}(q,p)+PV]} dq\; dp\; dV} \\ &= \frac{\frac{1}{N!}\frac{1}{h^{3N}} e^{-\beta[\mathcal{H}(q,p)+PV]}}{\frac{1}{N!}\frac{1}{h^{3N}} \int\int\int e^{-\beta[\mathcal{H}(q,p)+PV]} dq\; dp\; dV} \\ &= \frac{1}{Z}\frac{1}{N!}\frac{1}{h^{3N}} e^{-\beta[\mathcal{H}(q,p)+PV]}. \end{aligned} \tag{4}$$

Note that we used (2) in the last step. Combining 3 and 4, we can compute arbitrary ensemble averages $\langle X \rangle$ in the NPT ensemble:

$$\begin{aligned} \langle X \rangle &= \int\int\int X(q,p,V) f(q,p,V) dq\; dp\; dV \\ &= \frac{1}{Z}\frac{1}{N!}\frac{1}{h^{3N}} \int\int\int X(q,p,V) e^{-\beta[\mathcal{H}(q,p)+PV]} dq\; dp\; dV. \end{aligned} \tag{5}$$

Also, we will use one of the Maxwell relations

$$\left(\frac{\partial G}{\partial P}\right)_{N,T} = V, \tag{6}$$

and for the enthalpy, H,

$$H = -\left(\frac{\partial \ln(Z)}{\partial \beta}\right), \tag{7}$$

which can be derived from $H = U + PV = \langle \mathcal{H} + PV \rangle$.

By definition, the thermodynamic properties presented in this work are given as follows:

Isothermal compressibility κ

$$\kappa := -\frac{1}{V}\frac{\partial V}{\partial P}. \tag{8}$$

Heat capacity C_p

$$C_p = \frac{\partial H}{\partial T}. \tag{9}$$

Coefficient of thermal expansion α

$$\alpha = \frac{1}{V}\frac{\partial V}{\partial T}. \tag{10}$$

We start with the computation of κ. For this, we combine 6 and 1 with 8:

$$\kappa := -\frac{1}{V}\frac{\partial V}{\partial P} = -\frac{1}{V}\frac{\partial}{\partial P}\frac{\partial G}{\partial P} = -\frac{1}{V}\frac{\partial^2 G}{\partial P^2} = -\frac{1}{V}\frac{\partial^2}{\partial P^2}\left[-k_B T \ln(Z)\right].$$

This expression can be found by considering Z as a function of P:

$$
\begin{aligned}
\kappa &= -\frac{1}{V}\frac{\partial^2}{\partial P^2}\left[-k_B T \ln(Z)\right] \\
&= -\frac{1}{V}\frac{\partial}{\partial P}\left[-k_B T \frac{\partial}{\partial P}\ln(Z)\right] \\
&= -\frac{1}{V}\frac{\partial}{\partial P}\left[-k_B T \frac{1}{Z}\frac{\partial Z}{\partial P}\right] \\
&= \frac{k_B T}{V}\left[-\frac{1}{Z^2}\left(\frac{\partial Z}{\partial P}\right)^2 + \frac{1}{Z}\frac{\partial^2 Z}{\partial P^2}\right].
\end{aligned}
$$

Now, using the definition of Z (see equation (2)), we can determine the required partial derivatives of Z:

$$
\begin{aligned}
\frac{\partial Z}{\partial P} &= \frac{\partial}{\partial P}\frac{1}{N!}\frac{1}{h^{3N}}\int\int\int e^{-\beta[\mathcal{H}+PV]}dq\,dp\,dV \\
&= \frac{1}{N!}\frac{1}{h^{3N}}\int\int\int -\beta V e^{-\beta[\mathcal{H}+PV]}dq\,dp\,dV.
\end{aligned}
$$

Dividing this expression by the partition function then corresponds to an ensemble average given by (5) and therefore

$$
\begin{aligned}
\frac{\partial Z}{\partial P}\frac{1}{Z} &= \int\int\int -\beta V f(q,p,V)\,dq\,dp\,dV \\
&= -\beta\langle V\rangle.
\end{aligned}
\tag{11}
$$

The second derivative of Z with respect to P can be derived in the same manner:

$$
\begin{aligned}
\frac{\partial^2 Z}{\partial P^2} &= \frac{\partial^2}{\partial P^2}\frac{1}{N!}\frac{1}{h^{3N}}\int\int\int e^{-\beta[\mathcal{H}+PV]}dq\,dp\,dV \\
&= \frac{\partial}{\partial P}\frac{1}{N!}\frac{1}{h^{3N}}\int\int\int -\beta V e^{-\beta[\mathcal{H}+PV]}dq\,dp\,dV \\
&= \frac{1}{N!}\frac{1}{h^{3N}}\int\int\int \beta^2 V^2 e^{-\beta[\mathcal{H}+PV]}dq\,dp\,dV.
\end{aligned}
$$

Again, we can divide this expression by Z, yielding the required expression for an ensemble average:

$$
\begin{aligned}
\frac{\partial^2 Z}{\partial P^2}\frac{1}{Z} &= \int\int\int \beta^2 V^2 f(q,p,V)dq\, dp\, dV \\
&= \beta^2\langle V^2\rangle.
\end{aligned} \tag{12}
$$

Now, we can use (11) and (12) to find the final expression for κ:

$$
\begin{aligned}
\kappa &= \frac{k_B T}{V}\left[-\frac{1}{Z^2}\left(\frac{\partial Z}{\partial P}\right)^2 + \frac{1}{Z}\frac{\partial^2 Z}{\partial P^2}\right] \\
&= \frac{k_B T}{V}\left[-(-\beta\langle V\rangle)^2 + \beta^2\langle V^2\rangle\right] \\
&= \frac{k_B T}{V}\left[\beta^2(-\langle V\rangle^2 + \langle V^2\rangle)\right].
\end{aligned}
$$

Note that $\beta = \frac{1}{k_B T}$. Then:

$$
\kappa = \frac{1}{k_B T}\frac{1}{V}\left[\langle V^2\rangle - \langle V\rangle^2\right].
$$

Next, we derive a fluctuation formula for the heat capacity. For this purpose, we use the chain rule and equation (7). Since β is a function of the temperature T, we can rewrite C_p:

$$
\begin{aligned}
C_p &= \frac{\partial H}{\partial T} \\
&= \frac{\partial H}{\partial \beta}\frac{\partial \beta}{\partial T}.
\end{aligned}
$$

Then, using equation (7) and $\frac{\partial \beta}{\partial T} = -\frac{1}{k_B T^2}$, this can be again rewritten to

$$
\begin{aligned}
C_p &= \frac{\partial H}{\partial \beta}\frac{\partial \beta}{\partial T} \\
&= \frac{\partial}{\partial \beta}\left(-\frac{\partial \ln(Z)}{\partial \beta}\right)\left(-\frac{1}{k_B T^2}\right).
\end{aligned}
$$

Now, we can simplify this expression a bit further by computing the (partial) derivative of $\ln(Z)$:

$$
\begin{aligned}
C_p &= \frac{1}{k_B T^2}\frac{\partial}{\partial \beta}\left[\frac{1}{Z}\frac{\partial Z}{\partial \beta}\right] \\
&= \frac{1}{k_B T^2}\left[-\frac{1}{Z^2}\left(\frac{\partial Z}{\partial \beta}\right)^2 + \frac{1}{Z}\frac{\partial^2 Z}{\partial \beta^2}\right].
\end{aligned}
$$

Similar as above, we can again find an expression for C_p by computing partial derivatives of Z. The steps correspond to what has already been demonstrated above. We start by inserting the formal definition of Z and then compute the (partial) derivative of Z with respect to β:

$$
\begin{aligned}
\frac{\partial Z}{\partial \beta} &= \frac{\partial}{\partial \beta}\frac{1}{N!}\frac{1}{h^{3N}}\int\int\int e^{-\beta[\mathcal{H}+PV]}dq\, dp\, dV \\
&= \frac{1}{N!}\frac{1}{h^{3N}}\int\int\int -(\mathcal{H}+PV)e^{-\beta[\mathcal{H}+PV]}dq\, dp\, dV.
\end{aligned}
$$

With the enthalpy H being given by $H = \mathcal{H} + PV$ and dividing the expression above by Z, this gives us

$$
\frac{\partial Z}{\partial \beta}\frac{1}{Z} = -\langle H \rangle. \tag{13}
$$

In the same manner, we compute $\frac{\partial^2 Z}{\partial \beta^2}$:

$$\begin{aligned}
\frac{\partial^2 Z}{\partial \beta^2} &= \frac{\partial^2}{\partial \beta^2} \frac{1}{N!} \frac{1}{h^{3N}} \int \int \int e^{-\beta[\mathcal{H}+PV]} dq\, dp\, dV \\
&= \frac{\partial}{\partial \beta} \frac{1}{N!} \frac{1}{h^{3N}} \int \int \int -(\mathcal{H}+PV) e^{-\beta[\mathcal{H}+PV]} dq\, dp\, dV \\
&= \frac{1}{N!} \frac{1}{h^{3N}} \int \int \int (\mathcal{H}+PV)^2 e^{-\beta[\mathcal{H}+PV]} dq\, dp\, dV.
\end{aligned}$$

With this, and again $H = \mathcal{H} + PV$, the second required term for C_p corresponds to

$$\frac{\partial^2 Z}{\partial \beta^2} \frac{1}{Z} = \langle H^2 \rangle. \tag{14}$$

Combining (9) with (13) and (14), yields

$$\begin{aligned}
C_p &= \frac{1}{k_B T^2} \left[-\frac{1}{Z^2} \left(\frac{\partial Z}{\partial \beta} \right)^2 + \frac{1}{Z} \frac{\partial^2 Z}{\partial \beta^2} \right] \\
&= \frac{1}{k_B T^2} \left[-\langle H \rangle^2 + \langle H^2 \rangle \right],
\end{aligned}$$

which, after replacing k_B with the gas constant R appropriately, gives us the following expression for the heat capacity:

$$C_p = \frac{1}{NRT^2} (\langle H^2 \rangle - \langle H \rangle^2) = \frac{1}{NRT^2} \langle \Delta H^2 \rangle.$$

Finally, we try to find an expression for α (10). First, we apply the Maxwell relation (6):

$$\begin{aligned}\alpha &= \frac{1}{V}\frac{\partial V}{\partial T} \\ &= \frac{1}{V}\frac{\partial}{\partial T}\frac{\partial G}{\partial P} \\ &= \frac{1}{V}\frac{\partial^2 G}{\partial T \partial P}.\end{aligned}$$

Then, we apply the chain rule once again and use (1) to replace G:

$$\begin{aligned}\alpha &= \frac{1}{V}\frac{\partial^2 G}{\partial T \partial P} \\ &= \frac{1}{V}\frac{\partial^2 G}{\partial \beta \partial P}\frac{\partial \beta}{\partial T} \\ &= \frac{1}{V}\frac{\partial^2}{\partial \beta \partial P}(-k_B T \ln(Z))\frac{\partial \beta}{\partial T}.\end{aligned}$$

Again, with $\beta = \frac{1}{k_B T}$, it follows that $\frac{\partial \beta}{\partial T} = -\frac{1}{k_B T^2}$ and therefore

$$\begin{aligned}\alpha &= -\frac{1}{k_B T^2}\frac{1}{V}\frac{\partial^2}{\partial \beta \partial P}(-k_B T \ln(Z)) \\ &= \frac{1}{VT^2}\frac{\partial^2}{\partial \beta \partial P}(T \ln(Z)).\end{aligned}$$

Next, we apply some tricks: We rewrite T as an expression of β. With $\beta = \frac{1}{k_B T}$, it follows that $T = \frac{1}{k_B \beta}$. Moreover, we change the order of partial derivatives:

$$\begin{aligned}\alpha &= \frac{1}{VT^2}\frac{\partial^2}{\partial P \partial \beta}\left[T \ln(Z)\right] \\ &= \frac{1}{VT^2}\frac{\partial^2}{\partial P \partial \beta}\left[\frac{1}{k_B \beta}\ln(Z)\right] \\ &= \frac{1}{VT^2}\frac{\partial}{\partial P}\left[\frac{\partial}{\partial \beta}\left(\frac{1}{\beta k_B}\right)\ln(Z)\right].\end{aligned}$$

Note that Z is a function of β. That is, we have to apply the product rule:

$$\begin{aligned}\alpha &= \frac{1}{VT^2}\frac{\partial}{\partial P}\left[\frac{\partial}{\partial \beta}\left(\frac{1}{\beta k_B}\right)\ln(Z)\right] \\ &= \frac{1}{VT^2}\frac{\partial}{\partial P}\left[\left(-\frac{1}{\beta^2 k_B}\right)\ln(Z) + \left(\frac{1}{\beta k_B}\right)\frac{1}{Z}\frac{\partial Z}{\partial \beta}\right].\end{aligned}$$

Z is also a function of P. Hence, for the computation of the partial derivative with respect to the pressure P, we have to apply the product rule once again in the second term:

$$\alpha = \frac{1}{VT^2}\left[\left(-\frac{1}{\beta^2 k_B}\right)\frac{1}{Z}\frac{\partial Z}{\partial P} + \left(\frac{1}{\beta k_B}\right)\left(-\frac{1}{Z^2}\right)\frac{\partial Z}{\partial P}\frac{\partial Z}{\partial \beta} + \left(\frac{1}{\beta k_B}\right)\frac{1}{Z}\frac{\partial^2 Z}{\partial \beta \partial P}\right].$$

The first and second term are already known (see equation (11) and (13)). For the last part, we have to determine another second partial derivative of Z:

$$\begin{aligned}\frac{\partial^2 Z}{\partial P \partial \beta} &= \frac{\partial}{\partial P}\frac{1}{N!}\frac{1}{h^{3N}}\int\int\int -(\mathcal{H}+PV)e^{-\beta[\mathcal{H}+PV]}dq\;dp\;dV \\ &= \frac{1}{N!}\frac{1}{h^{3N}}\int\int\int\left(\frac{\partial}{\partial P}(-(\mathcal{H}+PV))\right)e^{-\beta[\mathcal{H}+PV]} \\ &+ (-(\mathcal{H}+PV))\left(\frac{\partial}{\partial P}e^{-\beta[\mathcal{H}+PV]}\right)dq\;dp\;dV \\ &= \frac{1}{N!}\frac{1}{h^{3N}}\int\int\int -V e^{-\beta[\mathcal{H}+PV]} + (-(\mathcal{H}+PV))(-\beta V)e^{-\beta[\mathcal{H}+PV]}dq\;dp\;dV.\end{aligned}$$

Dividing this expression by the partition function Z yields

$$\begin{aligned}\frac{1}{Z}\frac{\partial^2 Z}{\partial P \partial \beta} &= \int\int\int(-V+\beta VH)f(q,p,r)dq\;dp\;dV \\ &= \langle -V\rangle + \langle \beta VH\rangle.\end{aligned} \tag{15}$$

Now, we can combine (10) with (11), (13) and (15):

$$
\begin{aligned}
\alpha &= \frac{1}{VT^2}\left[\left(-\frac{1}{\beta^2 k_B}\right)\frac{1}{Z}\frac{\partial Z}{\partial P} + \left(\frac{1}{\beta k_B}\right)\left(-\frac{1}{Z^2}\right)\frac{\partial Z}{\partial P}\frac{\partial Z}{\partial \beta} + \left(\frac{1}{\beta k_B}\right)\frac{1}{Z}\frac{\partial^2 Z}{\partial \beta \partial P}\right] \\
&= \frac{1}{VT^2}\left[\left(-\frac{1}{\beta^2 k_B}\right)(-\beta\langle V\rangle) - \left(\frac{1}{\beta k_B}\right)(-\beta\langle V\rangle)(-\langle H\rangle) + \left(\frac{1}{\beta k_B}\right)(\langle -V\rangle + \langle \beta V H\rangle)\right] \\
&= \frac{1}{VT^2}\left[\left(\frac{1}{\beta k_B}\right)\langle V\rangle - \left(\frac{1}{k_B}\right)\langle V\rangle\langle H\rangle - \left(\frac{1}{\beta k_B}\right)\langle V\rangle + \left(\frac{1}{k_B}\right)\langle VH\rangle\right] \\
&= \frac{1}{VT^2}\left[-\left(\frac{1}{k_B}\right)\langle V\rangle\langle H\rangle + \left(\frac{1}{k_B}\right)\langle VH\rangle\right] \\
&= \frac{1}{VT^2}\left(\frac{1}{k_B}\right)\left[\langle VH\rangle - \langle V\rangle\langle H\rangle\right].
\end{aligned}
$$

Setting $V = \langle V\rangle$ and replacing k_B with the gas constant, we can obtain α as follows:

$$
\alpha = \frac{\langle VH\rangle - \langle H\rangle\langle V\rangle}{RT^2\langle V\rangle} = \frac{Cov(VH)}{RT^2\langle V\rangle},
$$

where $\langle Cov(VH)\rangle$ denotes the covariance of the box volume and the total energy.

To sum up, we obtained the following fluctuation formulas for κ, C_p and α:

Isothermal compressibility κ

$$
\kappa := -\frac{1}{V}\frac{\partial V}{\partial P} = \frac{\langle V^2\rangle - \langle V\rangle^2}{\langle V\rangle k_B T}. \tag{16}
$$

Heat capacity C_p

$$
C_p = \frac{\partial H}{\partial T} = \frac{\langle H^2\rangle - \langle H\rangle^2}{NRT^2}. \tag{17}
$$

Coefficient of thermal expansion α

$$
\alpha = \frac{1}{V}\frac{\partial V}{\partial T} = \frac{\langle VH\rangle - \langle H\rangle\langle V\rangle}{\langle V\rangle RT^2}. \tag{18}
$$

2 Additional Figures

2.1 MACE-OFF23(M) Hexane cavities

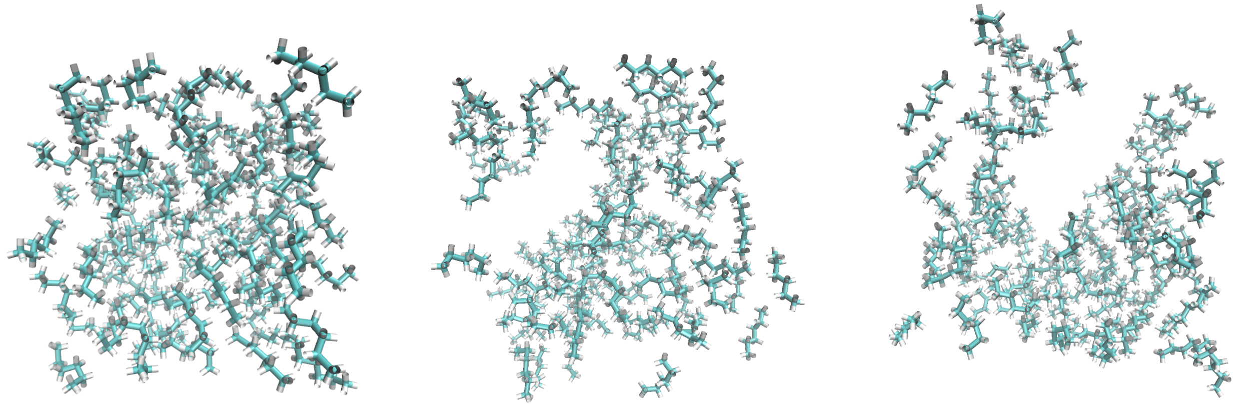

(a) Hexane box after FF equilibration.

(b) Hexane after 790ps production.

(c) Hexane after 890ps production.

Figure 1: Snapshots from a simulation of n-Hexane using MACE-OFF(M) in FP64.

2.2 Dependency of RDFs obtained with MACE on box volume

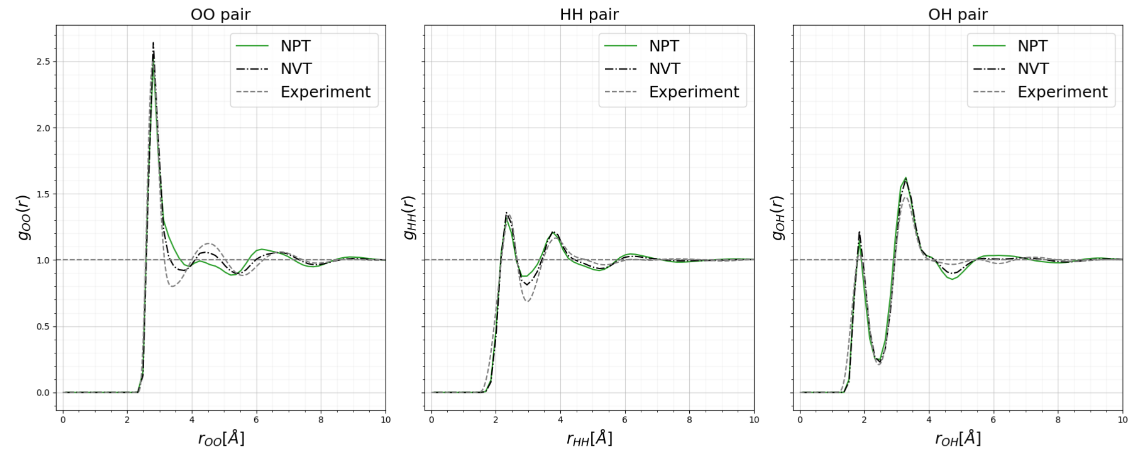

Figure 2: A comparison of MACE-OFF(S) RDFs of water computed from NVT and NPT ensembles. From left to right: O-O RDF, H-H RDF, O-H RDF for MACE-OFF23(S) for simulations run in the NVT ensemble (black dashed curve) and in the NPT ensemble (solid green line). Experimental RDFs shown as dashed, gray curves.

3 Summary of detailed results

The following tables (1) list all thermodynamic properties calculated. We present separately all properties obtained during the initial first 1 ns production simulation (cf. Methods), Tables 1–6, followed by the detailed results of the subsequent 5×1 ns simulations (Tables 7–12). (2) Table 13 lists the diffusion constants; (3) Tables 14 and 15 provide some performance information

3.1 Properties obtained from the initial 1 ns simulations

Tables 1–6 below contain no uncertainty estimate, since more reliable estimates can be obtained from the 5×1 ns simulations (see Tables 7–12).

Table 1: **Water.** Thermodynamic properties ΔH_{vap} (heat of vaporization), C_p (heat capacity), κ (isothermal compressibility), α (coefficient of thermal expansion) and ρ (density). Each value was computed as an ensemble average from a 1 ns simulation run in the NPT ensemble.

	$\Delta H_{vap}[kJ/mol]$	$C_p[cal/g/K]$	$\kappa[10^{-4}/bar]$	$\alpha[10^{-2}/K]$	$\rho[g/mL]$
Experiment	43.99	1	0.45	0.03	0.997
Force Field	43.49	1.06	0.52	0.10	1.01
ANI-2x	79.52	2.24	0.46	-0.22	1.00
MACE-OFF23(S)	47.79	1.64	0.26	0.09	1.12
MACE-OFF23(M)	48.90	1.57	0.23	0.10	1.18
MACE-OFF23(M)-FP32	48.90	1.52	0.23	0.09	1.18

Table 2: **Methanol.** Thermodynamic properties ΔH_{vap} (heat of vaporization), C_p (heat capacity), κ (isothermal compressibility), α (coefficient of thermal expansion) and ρ (density). Each value was computed as an ensemble average from a 1 ns simulation run in the NPT ensemble.

	$\Delta H_{vap}[kJ/mol]$	$C_p[cal/g/K]$	$\kappa[10^{-4}/bar]$	$\alpha[10^{-2}/K]$	$\rho[g/mL]$
Experiment	38.30	0.61	0.31	0.15	0.79
Force Field	38.29	1.47	0.94	0.14	0.77
ANI-2x	56.12	1.35	0.26	0.06	0.99
MACE-OFF23(S)	36.27	1.49	1.46	0.22	0.90
MACE-OFF23(M)	34.98	1.40	2.30	0.20	0.81

Table 3: **Acetone.** Thermodynamic properties ΔH_{vap} (heat of vaporization), C_p (heat capacity), κ (isothermal compressibility), α (coefficient of thermal expansion) and ρ (density). Each value was computed as an ensemble average from a 1 ns simulation run in the NPT ensemble.

	$\Delta H_{vap}[kJ/mol]$	$C_p[cal/g/K]$	$\kappa[10^{-4}/bar]$	$\alpha[10^{-2}/K]$	$\rho[g/mL]$
Experiment	31.90	0.52	1.41	0.15	0.78
Force Field	31.86	1.20	1.32	0.16	0.78
ANI-2x	26.05	1.37	2.53	0.29	0.82
MACE-OFF23(S)	29.92	1.20	1.35	0.19	0.88
MACE-OFF23(M)	20.05	1.48	29.09	1.10	0.64
MACE-OFF23(M)-FP32	20.78	1.23	8.48	0.48	0.66

Table 4: **NMA.** Thermodynamic properties ΔH_{vap} (heat of vaporization), C_p (heat capacity) κ (isothermal compressibility), α (coefficient of thermal expansion) and ρ (density). Each value was computed as an ensemble average from a 1 ns simulation run in the NPT ensemble.

	$\Delta H_{vap}[kJ/mol]$	$C_p[cal/g/K]$	$\kappa[10^{-4}/bar]$	$\alpha[10^{-2}/K]$	$\rho[g/mL]$
Experiment	53.50	NA	NA	NA	0.89
Force Field	55.41	1.23	0.81	0.12	0.90
ANI-2x	52.05	1.21	0.91	0.10	0.95
MACE-OFF23(S)	58.83	1.27	0.48	0.11	1.07
MACE-OFF23(M)	46.82	1.11	1.87	0.14	0.87

Table 5: **Benzene.** Thermodynamic properties ΔH_{vap} (heat of vaporization), C_p (heat capacity), κ (isothermal compressibility), α (coefficient of thermal expansion) and ρ (density). Each value was computed as an ensemble average from a 1 ns simulation run in the NPT ensemble.

	$\Delta H_{vap}[kJ/mol]$	$C_p[cal/g/K]$	$\kappa[10^{-4}/bar]$	$\alpha[10^{-2}/K]$	$\rho[g/mL]$
Experiment	33.83	0.42	0.97	0.11	0.88
Force Field	31.62	1.04	1.09	0.15	0.85
ANI-2x	34.01	1.06	0.63	0.1	0.93
MACE-OFF23(S)	36.27	1.26	0.71	0.16	1.02
MACE-OFF23(M)	28.11	1.24	1.97	0.27	0.89

Table 6: **n-Hexane.** Thermodynamic properties ΔH_{vap} (heat of vaporization), C_p (heat capacity), κ (isothermal compressibility), α (coefficient of thermal expansion) and ρ (density). Each value was computed as an ensemble average from a 1 ns simulation run in the NPT ensemble.

	$\Delta H_{vap}[kJ/mol]$	$C_p[cal/g/K]$	$\kappa[10^{-4}/bar]$	$\alpha[10^{-2}/K]$	$\rho[g/mL]$
Experiment	31.52	0.54	1.67	0.14	0.66
Force Field	32.14	1.55	1.91	0.20	0.65
ANI-2x	28.62	1.58	2.17	0.22	0.74
MACE-OFF23(S)	33.10	2.19	3.79	0.53	0.79
MACE-OFF23(M)	15.77	2.12	327.35	3.44	0.39
MACE-OFF23(M)-FP32	15.46	1.71	90.26	1.28	0.41

3.2 Properties obtained from the 5×1 ns simulations

Each of the Tables 7–12 below first reports the results for each individual run, followed by the mean and standard deviation of the five repeats (three repeats for MACE-OFF23(M) FP32).

Table 7: **Water.** From top to bottom: All condensed phase properties for all five force field runs, all five ANI-2x runs, all five MACE-OFF23(S) runs and all three MACE-OFF23(M) FP32 runs.

	$\Delta H_{vap}[kJ/mol]$	$C_p[cal/g/K]$	$\kappa[10^{-4}/bar]$	$\alpha[10^{-2}/K]$	$\rho[g/mL]$
Experiment	43.99	1.00	0.45	0.03	0.997
Force Field Run 1	43.49	1.03	0.54	0.09	1.01
Force Field Run 2	43.49	1.11	0.54	0.10	1.01
Force Field Run 3	43.49	1.13	0.55	0.09	1.01
Force Field Run 4	43.49	1.01	0.55	0.09	1.01
Force Field Run 5	43.48	1.01	0.57	0.10	1.01
Force Field Mean μ	43.49	1.06	0.55	0.09	1.01
Force Field Std Dev σ	0.00	0.06	0.01	0.01	0.00
ANI-2x Run 1	80.14	1.22	0.10	0.01	0.98
ANI-2x Run 2	80.22	1.17	0.13	0.00	0.98
ANI-2x Run 3	80.07	1.19	0.27	-0.02	0.99
ANI-2x Run 4	80.15	1.74	0.19	-0.08	0.99
ANI-2x Run 5	80.12	1.29	0.16	-0.03	0.98
ANI-2x Mean μ	80.14	1.32	0.17	-0.02	0.98
ANI-2x Std Dev σ	0.05	0.24	0.07	0.04	0.01
MACE-OFF(S) Run 1	47.80	1.79	0.30	0.12	1.12
MACE-OFF(S) Run 2	47.80	1.60	0.28	0.09	1.12
MACE-OFF(S) Run 3	47.78	1.69	0.30	0.11	1.12
MACE-OFF(S) Run 4	47.79	1.72	0.28	0.10	1.12
MACE-OFF(S) Run 5	47.83	1.76	0.29	0.10	1.12
MACE-OFF(S) Mean μ	47.80	1.71	0.29	0.10	1.12
MACE-OFF(S) Std Dev σ	0.02	0.07	0.01	0.01	0.00
MACE-OFF(M) FP32 Run 1	48.92	1.61	0.23	0.09	1.18
MACE-OFF(M) FP32 Run 2	48.92	1.45	0.24	0.08	1.18
MACE-OFF(M) FP32 Run 3	48.87	1.54	0.24	0.08	1.18
MACE-OFF(M) FP32 Mean μ	48.90	1.53	0.24	0.08	1.18
MACE-OFF(M) FP32 Std Dev σ	0.03	0.08	0.01	0.01	0.00

Table 8: **Methanol.** From top to bottom: All condensed phase properties for all five force field runs, all five ANI-2x runs and all five MACE-OFF23(S) runs.

	$\Delta H_{vap}[kJ/mol]$	$C_p[cal/g/K]$	$\kappa[10^{-4}/bar]$	$\alpha[10^{-2}/K]$	$\rho[g/mL]$
Experiment	38.30	0.61	1.31	0.15	0.79
Force Field Run 1	38.34	1.41	0.95	0.14	0.78
Force Field Run 2	38.29	1.35	0.95	0.14	0.77
Force Field Run 3	38.30	1.49	0.96	0.15	0.78
Force Field Run 4	38.31	1.26	0.91	0.12	0.78
Force Field Run 5	38.32	1.52	0.95	0.14	0.78
Force Field Mean μ	38.31	1.41	0.94	0.14	0.78
Force Field Std Dev σ	0.02	0.11	0.02	0.01	0.00
ANI-2x Run 1	55.99	1.42	0.30	0.09	0.99
ANI-2x Run 2	56.03	1.36	0.28	0.07	0.99
ANI-2x Run 3	56.04	1.39	0.26	0.07	0.99
ANI-2x Run 4	56.05	1.48	0.27	0.08	0.99
ANI-2x Run 5	56.01	1.54	0.32	0.09	0.99
ANI-2x Mean μ	56.02	1.44	0.29	0.08	0.99
ANI-2x Std Dev σ	0.02	0.07	0.02	0.01	0.00
MACE-OFF(S) Run 1	36.31	1.43	1.69	0.20	0.91
MACE-OFF(S) Run 2	36.31	1.54	1.27	0.22	0.91
MACE-OFF(S) Run 3	36.27	1.41	1.76	0.28	0.90
MACE-OFF(S) Run 4	36.23	1.32	1.54	0.18	0.90
MACE-OFF(S) Run 5	36.35	1.43	1.32	0.19	0.91
MACE-OFF(S) Mean μ	36.29	1.43	1.52	0.21	0.91
MACE-OFF(S) Std Dev σ	0.05	0.08	0.22	0.04	0.01

Table 9: **Acetone.** From top to bottom: All condensed phase properties for all five force field runs, all five ANI-2x runs, all five MACE-OFF23(S) runs and all three MACE-OFF23(M) FP32 runs.

	$\Delta H_{vap}[kJ/mol]$	$C_p[cal/g/K]$	$\kappa[10^{-4}/bar]$	$\alpha[10^{-2}/K]$	$\rho[g/mL]$
Experiment	31.90	0.52	1.41	0.15	0.78
Force Field Run 1	31.86	1.17	1.36	0.16	0.78
Force Field Run 2	31.85	1.20	1.33	0.15	0.78
Force Field Run 3	31.87	1.17	1.42	0.16	0.78
Force Field Run 4	31.78	1.17	1.52	0.19	0.78
Force Field Run 5	31.87	1.29	1.55	0.21	0.78
Force Field Mean μ	31.85	1.20	1.44	0.17	0.78
Force Field Std Dev σ	0.04	0.05	0.10	0.03	0.00
ANI-2x Run 1	26.09	1.16	1.67	0.15	0.82
ANI-2x Run 2	26.16	1.31	2.02	0.19	0.82
ANI-2x Run 3	26.17	1.26	1.92	0.22	0.82
ANI-2x Run 4	26.14	1.29	1.91	0.21	0.83
ANI-2x Run 5	26.18	1.14	1.89	0.21	0.83
ANI-2x Mean μ	26.15	1.23	1.88	0.20	0.82
ANI-2x Std Dev σ	0.04	0.08	0.13	0.03	0.01
MACE-OFF(S) Run 1	29.88	1.20	1.29	0.18	0.87
MACE-OFF(S) Run 2	29.94	1.23	1.10	0.16	0.88
MACE-OFF(S) Run 3	29.99	1.24	1.24	0.17	0.88
MACE-OFF(S) Run 4	29.91	1.25	1.41	0.19	0.88
MACE-OFF(S) Run 5	29.95	1.35	1.17	0.16	0.88
MACE-OFF(S) Mean μ	29.93	1.25	1.24	0.17	0.88
MACE-OFF(S) Std Dev σ	0.04	0.06	0.12	0.01	0.00
MACE-OFF(M) FP32 Run 1	20.69	1.22	13.59	0.54	0.66
MACE-OFF(M) FP32 Run 2	20.58	1.28	14.52	0.59	0.65
MACE-OFF(M) FP32 Run 3	20.44	1.39	21.82	0.82	0.64
MACE-OFF(M) FP32 Mean μ	20.57	1.30	16.64	0.65	0.65
MACE-OFF(M) FP32 Std Dev σ	0.13	0.09	4.51	0.15	0.01

Table 10: **NMA.** From top to bottom: All condensed phase properties for all five force field runs, all five ANI-2x runs and all five MACE-OFF23(S) runs.

	$\Delta H_{vap}[kJ/mol]$	$C_p[cal/g/K]$	$\kappa[10^{-4}/bar]$	$\alpha[10^{-2}/K]$	$\rho[g/mL]$
Experiment	53.50	NA	NA	NA	0.89
Force Field Run 1	55.46	1.16	0.78	0.11	0.90
Force Field Run 2	55.48	1.12	0.80	0.10	0.90
Force Field Run 3	55.39	1.07	0.75	0.10	0.90
Force Field Run 4	55.55	1.14	0.76	0.09	0.90
Force Field Run 5	55.40	1.14	0.76	0.10	0.90
Force Field Mean μ	55.46	1.13	0.77	0.10	0.90
Force Field Std Dev σ	0.07	0.03	0.02	0.01	0.00
ANI-2x Run 1	51.97	1.20	1.26	0.12	0.95
ANI-2x Run 2	52.04	1.10	1.01	0.11	0.95
ANI-2x Run 3	52.16	1.18	0.99	0.12	0.95
ANI-2x Run 4	52.15	1.14	1.10	0.13	0.95
ANI-2x Run 5	52.06	1.13	0.86	0.09	0.95
ANI-2x Mean μ	52.08	1.15	1.04	0.11	0.95
ANI-2x Std Dev σ	0.08	0.04	0.15	0.02	0.00
MACE-OFF(S) Run 1	58.90	1.18	0.46	0.09	1.07
MACE-OFF(S) Run 2	58.90	1.30	0.44	0.11	1.07
MACE-OFF(S) Run 3	58.96	1.22	0.40	0.09	1.07
MACE-OFF(S) Run 4	58.98	1.18	0.51	0.10	1.07
MACE-OFF(S) Run 5	58.82	1.31	0.55	0.12	1.07
MACE-OFF(S) Mean μ	58.91	1.24	0.47	0.10	1.07
MACE-OFF(S) Std Dev σ	0.06	0.06	0.06	0.01	0.00

Table 11: **Benzene.** From top to bottom: All condensed phase properties for all five force field runs, all five ANI-2x runs and all five MACE-OFF23(S) runs.

	$\Delta H_{vap}[kJ/mol]$	$C_p[cal/g/K]$	$\kappa[10^{-4}/bar]$	$\alpha[10^{-2}/K]$	$\rho[g/mL]$
Experiment	33.83	0.42	0.97	0.11	0.88
Force Field Run 1	31.64	1.06	1.24	0.16	0.84
Force Field Run 2	31.65	1.08	1.20	0.16	0.85
Force Field Run 3	31.66	1.06	1.11	0.14	0.85
Force Field Run 4	31.65	1.08	1.12	0.15	0.85
Force Field Run 5	31.64	1.05	1.15	0.15	0.84
Force Field Mean μ	31.65	1.07	1.16	0.15	0.85
Force Field Std Dev σ	0.01	0.01	0.06	0.01	0.01
ANI-2x Run 1	34.07	1.07	0.61	0.09	0.93
ANI-2x Run 2	34.03	1.05	0.58	0.07	0.93
ANI-2x Run 3	33.90	1.08	0.54	0.06	0.93
ANI-2x Run 4	34.00	1.05	0.63	0.08	0.93
ANI-2x Run 5	33.99	1.09	0.60	0.08	0.93
ANI-2x Mean μ	34.00	1.07	0.59	0.08	0.93
ANI-2x Std Dev σ	0.06	0.02	0.03	0.01	0.00
MACE-OFF(S) Run 1	35.98	1.21	0.69	0.14	1.02
MACE-OFF(S) Run 2	36.04	1.12	0.67	0.12	1.02
MACE-OFF(S) Run 3	36.31	1.21	0.80	0.19	1.02
MACE-OFF(S) Run 4	36.39	1.11	0.66	0.12	1.03
MACE-OFF(S) Run 5	36.22	1.25	0.76	0.17	1.02
MACE-OFF(S) Mean μ	36.19	1.18	0.72	0.15	1.02
MACE-OFF(S) Std Dev σ	0.17	0.06	0.06	0.03	0.00

Table 12: **n-Hexane.** From top to bottom: All condensed phase properties for all five force field runs, all five ANI-2x runs, all five MACE-OFF23(S) runs and all three MACE-OFF23(M) FP32 runs.

	$\Delta H_{vap}[kJ/mol]$	$C_p[cal/g/K]$	$\kappa[10^{-4}/bar]$	$\alpha[10^{-2}/K]$	$\rho[g/mL]$
Experiment	31.52	0.54	1.67	0.14	0.66
Force Field Run 1	32.26	1.65	1.70	0.16	0.65
Force Field Run 2	32.16	1.63	1.81	0.18	0.65
Force Field Run 3	32.21	1.55	2.07	0.20	0.65
Force Field Run 4	32.11	1.55	1.77	0.17	0.65
Force Field Run 5	32.07	1.61	1.86	0.16	0.65
Force Field Mean μ	32.16	1.60	1.84	0.17	0.65
Force Field Std Dev σ	0.08	0.05	0.14	0.02	0.00
ANI-2x Run 1	28.45	1.57	1.92	0.17	0.74
ANI-2x Run 2	28.45	1.68	1.98	0.23	0.74
ANI-2x Run 3	28.47	1.61	1.79	0.20	0.74
ANI-2x Run 4	28.35	1.80	2.30	0.25	0.74
ANI-2x Run 5	28.48	1.55	2.00	0.19	0.74
ANI-2x Mean μ	28.44	1.64	2.00	0.21	0.74
ANI-2x Std Dev σ	0.05	0.10	0.19	0.03	0.00
MACE-OFF(S) Run 1	32.61	1.93	2.64	0.35	0.78
MACE-OFF(S) Run 2	32.60	1.84	3.58	0.39	0.78
MACE-OFF(S) Run 3	32.34	2.29	5.05	0.66	0.77
MACE-OFF(S) Run 4	32.88	2.00	3.23	0.46	0.79
MACE-OFF(S) Run 5	32.53	2.37	7.70	0.80	0.78
MACE-OFF(S) Mean μ	32.59	2.09	4.44	0.53	0.78
MACE-OFF(S) Std Dev σ	0.19	0.23	2.03	0.19	0.01
MACE-OFF(M) FP32 Run 1	16.59	2.06	116.27	1.84	0.46
MACE-OFF(M) FP32 Run 2	16.71	1.80	46.45	0.91	0.47
MACE-OFF(M) FP32 Run 3	16.56	1.75	95.86	1.46	0.45
MACE-OFF(M) FP32 Mean μ	16.62	1.87	86.19	1.40	0.46
MACE-OFF(M) FP32 Std Dev σ	0.08	0.17	35.90	0.47	0.01

3.3 Diffusion constants from NVT simulations

Table 13: **Self diffusion coefficients** D $[10^{-9}m^2/s]$ for water, methanol, acetone, benzene and n-hexane.

D $[10^{-9}m^2/s]$	Water	Methanol	Acetone	Benzene	n-Hexane
Experiment	2.29	2.27	4.94	2.15	4.14
Force Field	5.43	2.28	3.17	1.41	4.08
ANI-2x	2.77×10^{-3}	5.11×10^{1}	1.06	2.44×10^{-1}	4.13×10^{-2}
MACE-OFF23(S)	2.20	5.75×10^{1}	1.14	2.06×10^{-1}	9.66×10^{-2}

3.4 Some representative timings

Finally, Tables 14 and 15 list the computational cost of the NNPs on the hardware available to us, using double precision (FP64) floating-point arithmetic. All simulations were carried out on Nvidia RTX4090 and RTX 6000 Ada devices. The MACE-OFF23(M) FP64 calculations required the ADA6000RTX cards with 48 GB of RAM; otherwise, the performance of the ADA6000 and RTX4090 cards is very similar.

Table 14: Computational performance in [ns / day] and ratios between methods. The timings are based on the last 100ps of NPT ensemble simulations using the Langevin integrator.

NPT	Water	Methanol	Acetone	Benzene	n-hexane	NMA	Mean	∼MM	∼ANI-2x	∼MACE(S)
N atoms	1716	1938	1780	1560	1960	1764				
MM	338	341	361	391	342	359	355.33	1		
ANI-2x	1.34	1.05	1.24	1.63	0.99	1.21	1.24	285.64	1	
MACE(S)	0.19	0.18	0.20	0.24	0.18	0.19	0.20	1791.60	6.27	1
MACE(M)	0.04	0.04	0.04	0.05	0.04	0.04	0.04	8741.29	30.60	4.88

Table 15: Computational performance in [ns / day] and ratios between methods. The timings are based on the last 100ps of NVT ensemble simulations using the Nosé-Hoover integrator.

NVT	Water	Methanol	Acetone	Benzene	n-hexane	Mean	∼MM	∼ANI-2x
N atoms	1716	1938	1780	1560	1960			
MM	142	142	146	147	140	143	1	
ANI-2x	0.74	0.58	0.67	0.87	0.60	0.69	207.17	1
MACE(S)	0.11	0.10	0.11	0.13	0.10	0.11	1314.15	6.34
